# Therapeutic potential of *Parabacteroides distasonis* in gastrointestinal and hepatic disease

**DOI:** 10.1002/mco2.70017

**Published:** 2024-12-16

**Authors:** Jingyi Duan, Qinmei Li, Yan Cheng, Weifeng Zhu, Hongning Liu, Fei Li

**Affiliations:** ^1^ Department of Gastroenterology & Hepatology Laboratory of Hepato‐intestinal Diseases and Metabolism Frontiers Science Center for Disease‐Related Molecular Network West China Hospital Sichuan University Chengdu China; ^2^ Deparment of Pharmacy, Academician Workstation Jiangxi University of Chinese Medicine Nanchang China; ^3^ Department of Gastroenterology & Hepatology, Huaxi Joint Centre for Gastrointestinal Cancer State Key Laboratory of Respiratory Health and Multimorbidity West China Hospital Sichuan University Chengdu China

**Keywords:** gastrointestinal and hepatic disease, metabolic pathways, natural components, *Parabacteroides distasonis*

## Abstract

Increasing evidences indicate that the gut microbiota is involved in the development and therapy of gastrointestinal and hepatic disease. Imbalance of gut microbiota occurs in the early stages of diseases, and maintaining the balance of the gut microbiota provides a new strategy for the treatment of diseases. It has been reported that *Parabacteroides distasonis* is associated with multiple diseases. As the next‐generation probiotics, several studies have demonstrated its positive regulation on the gastrointestinal and hepatic disease, including inflammatory bowel disease, colorectal cancer, hepatic fibrosis, and fatty liver. The function of *P. distasonis* and its metabolites mainly affect host immune system, intestinal barrier function, and metabolic networks. Manipulation of *P. distasonis* with natural components lead to the protective effect on enterohepatic disease. In this review, the metabolic pathways regulated by *P. distasonis* are summarized to illustrate its active metabolites and their impact on host metabolism, the role and action mechanism in gastrointestinal and hepatic disease are discussed. More importantly, the natural components can be used to manipulate *P. distasonis* as treatment strategies, and the challenges and perspectives of *P. distasonis* in clinical applications are discussed.

## Graphical abstract

This review illustrate the metabolic network medicated by *Parabacteroides distasonis* and its role and action mechanism in gastrointestinal and hepatic disease. The natural components affecting *Parabacteroides distasonis* was summarized, which show the therapeutic potential for gastrointestinal and hepatic diseases.

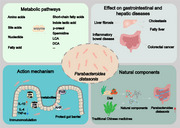

## 1 INTRODUCTION

Gastrointestinal and hepatic diseases such as luminal, liver, and pancreatic diseases pose serious threat to the quality of people's life and account for a significant portion of health care expenditures.^1^ Numerous factors could affect digestive system diseases, mainly genetic and environmental factors.^2^, ^3^ Environmental factors such as diet, obesity, lifestyle, and microbiota could lead to the onset and progression of disease.^4^, ^5^, ^6^, ^7^ Microbial genome sequencing of stool samples revealed significant changes of gut microbiota in fatty liver disease^8^ and inflammatory bowel disease (IBD).^9^

Intestine commensal microbiota plays a crucial role in host nutrient uptake, metabolism, establishment of mucosal immune system,^10^, ^11^ and help to construct intestinal epithelial barrier, defend against pathogens, support energy metabolism, and shape the immune system.^12^, ^13^ The secreted proteins, small molecular metabolites, or the bacteria themselves are directly or indirectly involved in the regulation of human health. For example, short‐chain fatty acids (SCFAs) and bile acids (BAs) produced by gut microbiota are able to affect host metabolism and immune function.^14^, ^15^ Imbalance of gut microbiota is closely related to intestinal tract and liver disease, and manipulating microbiota holds promise as an approach for treating them.^16^

*Parabacteroides distasonis* was first isolated from human stools and classified as a member of the genus *Bacteroides*.^17^ However, analysis of its 16S rRNA sequence indicated that 90% gene sequence similarity to *Tannerella forsythensis*.^18^ Additionally, the major menaquinones found in *P. distasonis* are MK‐9 and MK‐10, which are different from those typically found in *Bacteroides* species.^18^ Based on these characteristics, *P. distasonis* has been reclassified as the genus *Parabacteroides* and the family *Tannerellaceae*. It is recognized as the type species of genus *Parabacteroides*.^18^ Noteworthily, *P. distasonis* shows the protective effects against diseases such as obesity^19^ and colitis^20^ and is expected to be one of the next‐generation probiotics.^21^ Recent studies reported that *P. distasonis* regulated host metabolism,^19^ gut barrier, and immunity.^22^, ^23^ Its metabolites are mainly classified into SCFAs, amino acid, BAs, and other active compounds, which contribute to various physiological processes.^24^

Multiple studies have demonstrated that *P. distasonis* plays an important role in diseases. Understanding its ability to generate metabolites and action mechanism is able to better facilitate utilization of this bacteria. However, the relationship between *P. distasonis*, gastrointestinal and hepatic disease, and the treatment strategies has not been comprehensively discussed in previous study. In this review, we focused on the reported metabolic pathways of *P. distasonis* and its metabolites both in vivo and in vitro. We mainly discussed the role of *P. distasonis* in gastrointestinal and hepatic disease and its mechanisms of immune and metabolic regulation. We also summarized the natural components that are able to regulate *P. distasonis*, which could be potentially used as effective agents for diseases treatment. Additionally, clinical trials related to *P. distasonis* mainly through dietary interventions and prebiotics to regulate its abundance have been summarized. Finally, we discussed the prospect and limitations of its clinical application.

## 2 FEATURES OF *P. DISTASONIS*

*P. distasonis* exhibits a protective role in various diseases, but has been also observed negatively involved in some infectious diseases. The dichotomous role of *P. distasonis* may be attributed to strain variability. Here, we discuss the features of *P. distasonis* including the basic structural composition, utilization of polysaccharides, antibiotic resistance and biofilm formation. These features are associated with the colonization, adhesion, and invasion of *P. distasonis* in the host.

### 2.1 Characteristics of structure

*P. distasonis* belongs to gram‐negative anaerobic bacteria. Its structure is consistent with the structural characteristics of gram‐negative bacteria. Lipopolysaccharides (LPS) is the main component of outer membrane of gram‐negative bacteria. In the lipid portion of LPS from strain ATCC 8503, large amounts of anteisobranched pentadecanoic acid were found, while 3‐hydroxyheptadecanoic acid was not detected.^25^ O‐antigen is a component of LPS, which is related to bacterial virulence. The *rfbA* gene encoding the enzyme that catalyzes the formation of O‐antigen in the initial step, and the variation in this gene may be used to classify different strains of *P. distasonis*.^26^ The production and structure of O‐antigen may help us distinguish pathogenic strains in *P. distasonis*, but more experimental evidence is needed.

The structure of outer‐membrane protein (OMP) contributes to bacterial resistance. An OMP was detected from a clinical strain of *P. distasonis*. Test of pore‐forming activity in liposomes indicates that this protein may form pore.^27^ It has the characteristic of OmpA protein, and the *ompA* gene was identified in *P. distasonis*.^28^ Further studies are needed to elucidate the impact of OMP on *P. distasonis* resistance and its variation among resistant strains.

Nine glycoproteins have been identified as surface layer (S‐layer) glycoproteins on the *P. distasonis*.^29^ The composition of glycoproteins is the result of its adaptation to the host environment, which may contribute to its own survival. The function of S‐layer glycoproteins is still unclear. Previous studies have demonstrated that S‐layer proteins is able to contribute to adhesion,^30^ immune response,^31^ and the form of biofilm.^32^, ^33^ Those may render some *P. distasonis* strains to adhere and invade cells.

Capsules are the outermost structures of *Bacteroides fragilis*, which are composed of acidic polyanionic exopolymer.^34^ Encapsulated bacteria have enhanced ability to adhere to rat peritoneal mesothelium^35^ and resist host phagocytosis,^36^ which is related to the enhancement of virulence. Capsules were also detected in other *Bacteroides* species, and *P. distasonis* has been found not to be encapsulated through India ink wet mounts, electron microscopy, and ruthenium red staining.^37^, ^38^ However, the clinically isolated strains of *P. distasonis* have been observed with capsules structures by transmission electron microscopy.^39^ This indicated that some strains of *P. distasonis* have capsules, which may be related to their pathogenicity.

### 2.2 Utilization of polysaccharides

*P. distasonis* ATCC 8503 is the typical strain. The complete genome sequences of *P. distasonis* ATCC 8503 describes its metabolic profile. Gene Ontology analysis shows that polysaccharide metabolism, protein degradation, and cofactor biosynthesis are the main metabolic pathways.^40^ Polysaccharides are one of the nutritional sources for *P. distasonis*, and the utilization of polysaccharides helps the colonization of *P. distasonis* in the gut. Here is a summary of the ability of *P. distasonis* to utilize polysaccharides and the enzymes involved.

Compared with other *Bacteroidetes* in the gut, *P. distasonis* has the minimal number of genes involved in carbon source degradation. A total of 97 glycoside hydrolases belonging to 31 glycoside hydrolases families (as described in Carbohydrate‐Active Enzymes database) were identified.^40^ *P. distasonis* ferments carbohydrates such as galactose, inulin, raffinose, and trehalose, but lacks the ability to break down l‐arabinose, glycerin, and cellulose.^17^, ^18^, ^40^ Mucopolysaccharidase was achieved from a clinical separated strain of *P. distasonis*, hyaluronidase and chondroitinase like activity was detected in the purified enzyme.^41^ Neuraminidase belongs to glycoside hydrolase, metabolizes glycoconjugates, and removes sialic acids.^42^ Three strains of *P. distasonis* are positive for neuraminidase assay, when cultured in digest broth and 5% proteose peptone water broth.^43^ The result of genome sequencing also showed that *P. distasonis* has polysaccharide deacetylases and enzymes to degrade proteins, but lack polysaccharide lyases.^40^

Colonic mucus layer is mainly composed of mucin *O*‐glycan, and the relative abundance of *P. distasonis* is correlated with mucin *O*‐glycans decorated with terminal fucose.^44^ *P. distasonis* possess α‐fucosidases to utilize l‐fucose and may degrade mucin *O*‐glycans as carbon source.^40^, ^45^ Additionally, *P. distasonis* forms fucosylated glycan decorated glycoproteins, which is able to enhance the colonization ability of microorganisms.^46^, ^47^

The similar metabolic pathway of *N*‐glycan was found in *P. distasonis*. Glycoside hydrolase in *P. distasonis* degrades *N*‐glycan to β‐1,4‐d‐mannosyl‐N‐acetyl‐d‐glucosamine, which may be phosphorylated by a specific enzyme and directly enter to glycolysis.^48^ Apart from 2‐β‐d‐glucooligosaccharide sophorohydrolase, laminarinase and mucopolysacharidase were obtained from *P. distasonis*.^41^, ^49^, ^50^ The final products of fermentation include succinic acid, acetic acid, propionic acid, and isovaleric acid.^51^ The metagenomic data have revealed that *P. distasonis* codes phosphotransacetylase, an enzyme that is capable of producing acetic acid.^52^

Overall, *P. distasonis* has various glycoside hydrolases which degrade substances that are difficult to degrade by the host. In this process, *P. distasonis* produces small molecules that are capable of influencing the intestinal environment. In addition, polysaccharides are also used to stimulate the growth of *P. distasonis* and exert therapeutic effects on diseases.

### 2.3 Antibiotic resistance

*P. distasonis* was the most resistant species in the isolated *B. fragilis* group when exposed to penicillin, cefoxitin, and cefotaxime.^53^ Among 2673 isolated *B. fragilis* group, *P. distasonis* exhibited the highest resistance to beta‐lactam antibiotics.^54^ The resistant strains express beta‐lactamase.^53^ The genes encoding this enzyme including *bla*_TEM‐1_, *bla*_BIL‐1_,^55^ *cepA*,^56^ *cfiA*,^56^ and a unique gene similarity with *cfxA* and *cfxA2* ^57^ were detected in *P. distasonis*.

However, clavulanic acid, the inhibitor of beta‐lactamase, cannot change the susceptibility of *P. distasonis* to cefamandole, cefoxitin, and cephalothin. On the other hand, ethylenediaminetetraacetate enhances the barrier permeability, resulting in increased minimal inhibitory concentrations of beta‐lactam antibiotics. The combination of clavulanic acid and ethylenediaminetetraacetate shows a synergistic effect, indicating that lower permeability is the major factor in producing resistantance.^58^ The components of penicillin‐binding proteins (PBPs) were reduced, absent, or altered in molecular weight in resistant strains. This alteration in PBPs is correlated to cefoxitin resistance.^59^

The percentage of clindamycin, amoxicillin/clavulanic acid, and imipenem‐resistant strains were increased in recent years.^60^ The resistance to clindamycin, erythromycin, streptogramins, and tetracycline in a strain of clinically isolated *P. distasonis* is able to be transferred to *B. fragilis*, indicating that the antibiotic resistance is specifically mediated by plasmid.^61^ The resistance to clindamycin was correlated with the erythromycin ribosomal methylase F (*ermF*) gene, which encodes methyltransferase.^62^ The *tetQ* gene, which encodes protein to protect bacteria from tetracycline, has been detected in *P. distasonis* 8503 through PCR assay, and the *tetQ* gene may be transferred to *Enterococcus faecalis*.^63^ The analysis of antibiotic resistance and resistance genes in different strains of *P. distasonis* is one aspect of safety assessment. It is also necessary to evaluate the impact of transferred antibiotic resistance genes on the host.

### 2.4 Formation of biofilm

In the process of biofilm formation, the planktonic bacteria initially adhere to surface by physicochemical processes. Then, the bacteria secrete extracellular polymeric substances, which mainly contains polysaccharides, lipid, and enzymes. This secretion leads to the adhesion from reversible to irreversible.^64^ In a study, microscopic observation was used to evaluate the biofilm formation capacities. All 14 strains of *P. distasonis* have the ability to form biofilm, although the biofilm structure is different due to its variation.^33^ Chronic stress reduces the abundance of *P. distasonis* in the cecum^65^; the stress‐induced molecules are one of the factors that affect the biofilm formation, and most molecules reduce the biofilm formation capacity.^33^ Interestingly, the natural component celastrol was reported to promote the biofilms formation of *P. distasonis* and increase its abundance in cecum.^66^ Biofilms formation may also affect the abundance of *P. distasonis* in the intestine, but there is still limited research on the substances and mechanisms that stimulate biofilms formation.

In general, antibiotic resistance, the presence of capsules, and the biofilm structure are related to the strain of *P. distasonis*. These characteristics not only affected the colonization of *P. distasonis* in the intestine, but also were relate to its potential for invasion and infection within the body. Further investigation into the relationship between strains and these characteristics will provide valuable direction for the secure research on *P. distasonis*.

## 3 METABOLIC PATHWAYS REGULATED BY *P. DISTASONIS*

**FIGURE 1:**
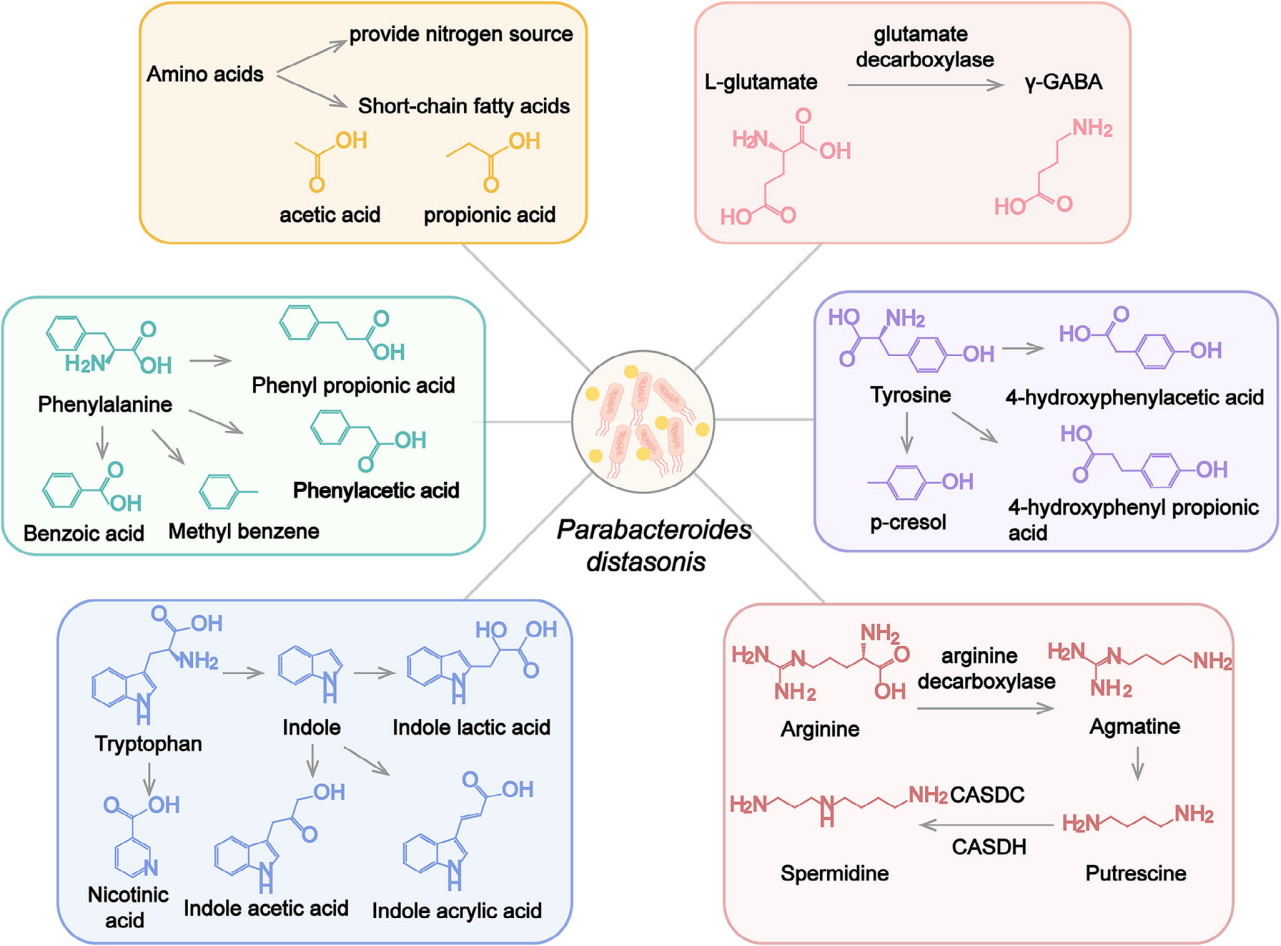
*P. distasonis* regulates amino acids metabolism and produce bioactive metabolites. During the decomposition process, *P. distasonis* is able to utilize amino acids as energy sources and generates small bioactive molecules that have important impacts on the host. Short‐chain fatty acids are the final products of amino acid metabolism. *P. distasonis* mainly produces acetic acid and propionic acid. *P. distasonis* metabolizes l‐glutamate to γ‐GABA via glutamate decarboxylase. *P. distasonis* is able to metabolize aromatic amino acids (phenylalanine, tyrosine, and tryptophan) and arginine to produce corresponding metabolites. However, the enzymes involved in this process are not yet been elucidated. γ‐GABA, gamma‐aminobutyric acid; CASDC, carboxyspermidine decarboxylase; CASDH, carboxyspermidine dehydrogenase.

The active metabolites produced by gut microbiota are able to directly affect intestinal function or regulate physiopathological processes by circulating to other organs.^67^ Multiple studies have found that *P. distasonis* generates various metabolites to participate in the regulation of the body. Metabolomic data have provided a more comprehensive understanding of its metabolic capacity. Limitations by the functional annotation of metabolic enzymes and the actual metabolites of *P. distasonis* still require experimental verification. Based on these premises, we have compiled literatures on the metabolites affected by *P. distasonis* and analyzed its metabolic capacity (Figure 1). This section focuses on the metabolites that may be produced in vivo or in vitro.

### 3.1 Amino acid metabolism

Gut microbiota play an important role in amino acid metabolism. Proteins from the diet are hydrolyzed by microbiota into amino acids and peptides, which further absorbed by the host or bacteria.^68^ Amino acids and peptides are able to enter bacteria cells through specific transport proteins, participate in protein synthesis, or be metabolized to small molecules. *P. distasonis* is capable of utilizing most amino acids and dipeptides for self‐reproduction or producing active molecules. For example, asparagine may be used as the sole nitrogen sources for *P. distasonis*, and the related genes (*ansA* and *aspA*) are found in its genomes.^69^ Amino acids are degraded to small bioactive molecules through multiple steps such as decarboxylation and deamination.^68^ Gamma‐aminobutyric acid (γ‐GABA) is an inhibitory neurotransmitter. *P. distasonis* decarboxylates l‐glutamate to γ‐GABA by glutamate decarboxylase,^70^ which may affect neuroendocrine in female.^71^

SCFAs are not only derived from the decomposition of carbohydrates, but also come from the metabolism of amino acids. Amino acids are transformed to corresponding keto acids or saturated fatty acids through transamination or deamination reaction by bacteria. SCFAs are generated as final products through a series of steps,^68^, ^72^ *P. distasonis* mainly produces acetic acid and propionic acid in this process.^73^, ^74^

*P. distasonis* has the ability to metabolize aromatic amino acids in the presence or absence of glucose.^75^ *P. distasonis* transforms phenylacetic acid, benzoic acid, phenyl propionic acid, and methyl benzene from phenylalanine.^75^ The supplement of glucose promotes the production of phenyl pyruvic acid and phenyl lactic acid, which are  the oxidative products of phenylalanine.^75^ Tyrosine may be decomposed into 4‐hydroxyphenylacetic acid and 4‐hydroxyphenyl propionic acid by *P. distasonis*. Furthermore, *p*‐cresol, a bioactive microbial metabolite from tyrosine, has been detected when *P. distasonis* was cultured in yeast extract, casitone, and fatty acid (YCFA) medium.^75^ However, in another report, no *p*‐cresol was detected in medium when *P. distasonis* was cultured in modified Gifu anaerobic medium.^76^ This process may be influenced by other substances in culture medium. Tryptophan only generates indole and indole derivatives under the action of bacteria.^77^, ^78^ Tryptophan is metabolized by *P. distasonis* to indole,^73^ indole‐3‐acetic acid,^75^ indolelactic acid,^66^, ^69^, ^75^, ^79^ and indoleacrylic acid,^80^ while indole was not detected in *P. distasonis* cultured in brain heart infusion (BHI) medium in another study.^51^ Nicotinic acid (NA) is also the product of tryptophan metabolism by gut microbiota. Quinolinate, which is derived from tryptophan metabolism, serves as a precursor substance in the synthesis of NA.^81^ The data of genome sequencing indicate that *P. distasonis* possesses the genes for NA synthesis. *P. distasonis* carries genes encoding nicotinate‐nucleotide pyrophosphorylase, 2′,3′‐cyclic nucleotide 2′‐phosphodiesterase, and purine nucleoside phosphorylase, which are involved in the pathway from quinolinate to NA. After administering *P. distasonis* to mice, the levels of NA in feces were increased, and the increased NA has been detected in multiple *P. distasonis* strains cultured in vitro. These findings indicated that *P. distasonis* indeed has the ability to generate NA. However, *P. distasonis* cannot increase the level of nicotinamide.^82^

The different results of those in vitro experiments may be attributed to differences in strain diversity, culture medium, and detection sensitivity. In summary, *P. distasonis* utilizes multiple amino acids and metabolize amino acids into small bioactive molecules. Among them, *p*‐cresol,^83^ indole,^84^ indole‐3‐acetic acid,^84^ indole lactic acid,^85^ and indole acrylic acid^80^ are natural ligands of aryl hydrocarbon receptor (AhR). The AhR pathway affects the integrity of the intestinal barrier.^86^ In addition, SCFAs and NA have been found to exhibit a protective effect on the intestinal barrier, which may be achieved through G protein coupled receptors. Those metabolites may relate to the function of *P. distasonis* and contribute to the function of *P. distasonis* in maintaining intestinal barrier integrity.

### 3.2 Polyamine metabolism

Polyamines, mainly including putrescine and spermidine, belong to small polycationic molecules. Many bacteria have the ability to produce, utilize or degrade polyamines.^87^ This process is common reaction in bacteria and may be related to their classification.^87^, ^88^ The distribution of polyamines may serve as a chemotaxonomic marker in bacterial classification.^88^ Strains in phylum *Bacteroidetes* were cultured in anaerobic environment to evaluate the level of polyamines by high‐performance liquid chromatography. It was found that spermidine is the predominant polyamine in *Parabacteroides*.^89^

It is reported that arginine decarboxylation is the dominant pathway for the biosynthesis of polyamines.^90^ Arginine may be converted into putrescine through either the arginine deiminase or arginine decarboxylase catalyzed pathway, with ornithine or agmatine serving as intermediates, respectively.^91^ In a metabolomics analysis of gut microbiota, the arginine levels decreased and agmatine levels increased in the culture medium of *P. distasonis*,^69^ which may be related to the biosynthesis of polyamines.^92^ Compared with reported polyamine biosynthetic and transport proteins, *P. distasonis* contains genes that encode homologous proteins of agmatine deiminase and *N*‐carbamoylputrescine amidohydrolase in putrescine synthesis.^93^ The homologs of carboxyspermidine decarboxylase and carboxyspermidine dehydrogenase metabolize putrescine into spermidine.^93^ Putrescine, spermine, and spermidine were found in the cell of *P. distasonis*.^89^, ^93^ The levels of polyamines in the body are influenced by the gut microbiota.^94^ Transplantation of *P. distasonis* increased the spermine and putrescine and reduced *N*‐acetylspermidine in the cecum of mice.^95^ This further demonstrates the ability of *P. distasonis* to regulate polyamine metabolism.

### 3.3 Nucleotide and fatty acid metabolism

Nucleotide metabolism is the other major metabolic pathway influenced by *P. distasonis*. It has been observed that *P. distasonis* increases the level of uracil in polyamine‐free media,^69^ mega media,^69^ and BHI medium.^66^ Uracil from microbiota stimulates chronic inflammation in the intestine and activate innate immunity response.^96^ This may explain the regulatory effects of *P. distasonis* on intestinal inflammation and immunity.

Bacteria are the main source of exogenous purines in the gut, which promotes mucous barrier integrity.^97^ *P. distasonis* increases the levels of xanthosine, xanthine, hypoxanthine, and guanine and decreases the levels of adenine and adenosine.^66^, ^69^ In nucleotide metabolism pathway, adenine and adenosine serve as upstream components that may provide raw materials for the synthesis of hypoxanthine, xanthosine, xanthine, and guanine. Supplementing intestinal hypoxanthine restores energy metabolism disorders caused by inflammation and improves barrier function.^98^ This suggested that the nucleotide metabolism regulated by *P. distasonis* may contribute to its beneficial effects on the intestine.

Gut microbiota also participates in fatty acid metabolism, and the changes in microbiota affect the composition of lipid in the host. *P. distasonis* participates in the metabolism of polyunsaturated fatty acids. γ‐Linolenic acid was increased in the cecum and liver after the transplantation of live *P. distasonis*.^99^ *P. distasonis* produces γ‐linolenic acid in high‐fat diet (HFD) extract, indicating its ability to metabolize fatty acids.^99^ The regulation of fatty acid metabolism by *P. distasonis* may be one of the mechanisms for treating metabolic diseases, but there is currently limited research on the metabolites of fatty acid.

### 3.4 BA metabolism

BAs are synthesized in the liver from cholesterol and modified by microbiota in the distal gastrointestinal tract. Conjugated and deconjugated BAs are absorbed to blood through BA transporter or passive diffusion in the enterohepatic circulation. BAs function as signaling molecules regulating many important physiological processes including metabolism^100^ and immune responses.^101^ BAs conjugated with taurine or glycine are secreted into small intestine and converted to secondary BAs in distal ileum, cecum, and colon through deconjugation, dehydrogenation, dihydroxylation and epimerization.^102^, ^103^ Gut microbiota is necessary for the hydrolysis of conjugated BAs and biotransformation of free BAs.

Recent studies have identified several enzymes involved in shaping BA pool in *P. distasonis*. Target BAs metabolomics and RNA‐Seq analysis revealed that *P. distasonis* possesses bile salt hydrolase (BSH),^104^ an enzyme deconjugating the conjugated BAs. BSH controls a crucial step in the conversion of BAs transformation in intestine and is essential for maintaining BAs homeostasis in the host. *P. distasonis* isolated from human has the ability to deconjugate taurocholic acid in vitro and vivo through BSH.^105^ The administration of *P. distasonis* alleviates hepatic fibrosis by increasing the activity of BSH and reducing the level of taurochenodeoxycholic acid (TCDCA), which induces pyroptosis in the liver.^66^

The deconjugated BAs further transform to new BAs in intestine. In a study, no secondary BAs were detected in the cecum of germ‐free mice‐inoculated *P. distasonis* strain K‐5 the mainly BAs were β‐muricholic acid (β‐MCA). Additionally, the results in vitro showed that *P. distasonis* deconjugated tauro‐β‐muricholic acid (T‐β‐MCA) but lacked 7α‐dehydroxylating activity.^106^ Interestingly, strain K‐5 and its extract were able to stimulate 7α‐dehydroxylation and 7β‐dehydroxylation, which expressed by *Eubacterium* sp. strain 36S.^107^

*P. distasonis* produced deoxycholic acid (DCA) and 7‐oxodeoxycholic acid (7‐oxoDCA) from cholic acid (CA) when sodium taurocholate served as a substrate in YCFA medium.^19^ With the supplement of sodium TCDCA, TCDCA undergoes hydrolysis to produce chenodeoxycholic acid (CDCA), then CDCA is converted to ursodeoxycholic acid (UDCA) through 7‐oxolithocholic acid (7‐oxoLCA).^19^ 7α‐Hydroxysteroid dehydrogenase (7α‐HSDH) and 7β‐hydroxysteroid dehydrogenase (7β‐HSDH) are known to catalyze these processes^108^ and may participate in the production of UDCA. Live *P. distasonis* increased the levels of UDCA, glycoursodeoxycholic acid, lithocholic acid (LCA), and taurolithocholic acid (TLCA) in the cecum of mice with calorie restriction diet or HFD.^109^

3‐Oxolithocholic acid (3‐oxoLCA) and isolithocholic acid (isoLCA), the isoforms of LCA, have been identified as inhibitors of T helper 17 cells (Th17) differentiation.^110^, ^111^ The synthesis of 3‐oxoLCA and isoLCA is associated with gut microbiota.^111^ Recent research has clarified that *P. distasonis* generated LCA, 3‐oxoLCA, and isoLCA through 5β‐reductase, 3α‐hydroxysteroid dehydrogenase (3α‐HSDH), and 3β‐hydroxysteroid dehydrogenase (3β‐HSDH) in vitro.^112^ *P. distasonis* produced 3‐oxoLCA from 3‐oxo‐Δ^4^‐LCA and converted 3‐oxoLCA, isoLCA, and LCA into each other through the action of 3α‐HSDH and 3β‐HSDH.^112^ The levels of 3‐oxoLCA, isoLCA, LCA, and DCA were increased in mice treated with live *P. distasonis*.^113^ The transplantation of *P. distasonis* ameliorated rheumatoid arthritis and inhibited the generation of 3‐oxoLCA, isoLCA, LCA, and DCA,^113^ indicating that the change of BA pool is the key for the function of *P. distasonis*. The latest research has found that *P. distasonis* converted CA into a newly detected BA called 3‐acetylcholic acid in vitro.^114^

The BAs produced by *P. distasonis* are related to the raw materials provided in vitro, which may lead to different production of BAs in vivo and in vitro. *P. distasonis* is able to utilize BAs from other bacteria to produce new BAs in the gut. Thereby, its regulation of BA pool also involves the interaction with other gut microbiota, which is also worth exploring.

**FIGURE 2:**
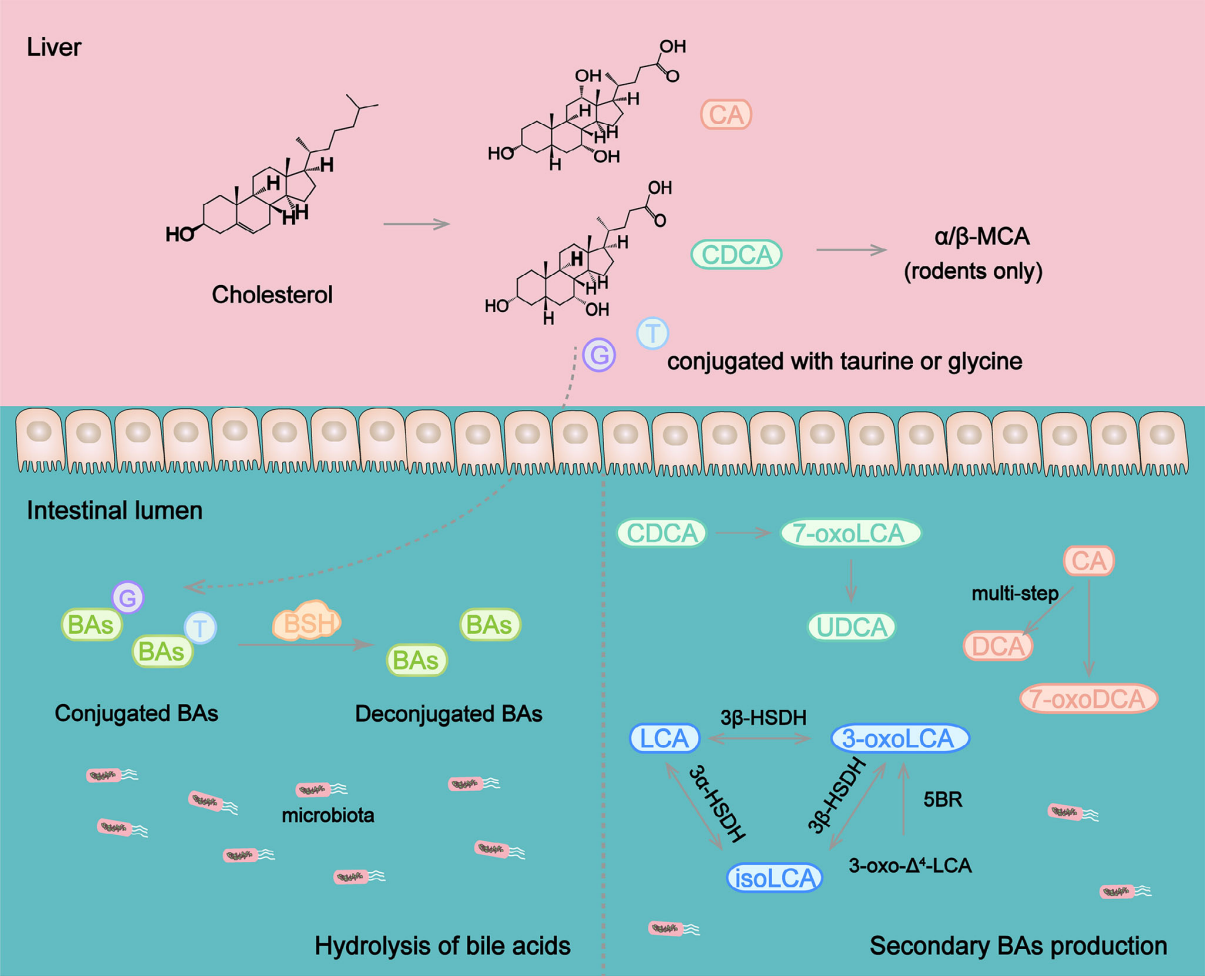
*P. distasonis* participates in bile acid metabolism. Primary bile acids are synthesized from cholesterol in the liver; CA and CDCA are the primary bile acids in humans. CDCA is only converted to MCA in rodents. After binding with glycine or taurine, free primary bile acids enter the intestine. *P. distasonis* possesses the BSH, which hydrolyzes conjugated bile acids. Free bile acids are further metabolized into secondary bile acids. CDCA is able to be metabolized to UDCA by *P. distasonis*. When CA is used as a substrate, *P. distasonis* is able to metabolize it to produce DCA and 7‐oxoDCA. Furthermore, *P. distasonis* possesses enzymes that interconvert LCA, isoLCA, and 3‐oxoLCA. CA, cholic acid; CDCA, chenodeoxycholic acid; BAs, bile acids; BSH, bile salt hydrolase; UDCA, ursodeoxycholic acid; DCA, deoxycholic acid; LCA, lithocholic acid; 3α‐HSDH, 3α‐hydroxysteroid dehydrogenase; 3β‐HSDH, 3β‐hydroxysteroid dehydrogenase; 5BR, 5β‐reductase.

Overall, researches on *P. distasonis* have focused on its impact on BA metabolism (Figure 2), as its production of active metabolites that affect various physiological processes. It is worth noting that its metabolic capability is not limited to those reported. Limited by culture conditions or detection techniques, there may be additional metabolites yet to be explored. In addition, its effect on the metabolites is not only limited to the metabolism of substances by its own metabolic enzymes, but also involves the cometabolic effects with other intestinal microbiota. It may be the reason why part results of in vivo and in vitro experiments are not consistent.

## 4 ROLE OF *P. DISTASONIS* IN GASTROINTESTINAL AND HEPATIC DISEASE

Dysbiosis of the gut microbiome is associated with many systemic diseases. More attention has been paid to the connection between *P. distasonis* and diseases, especially for gastrointestinal and hepatic disease. Here, we conducted a literature search for recent reports on the treatment of *P. distasonis* (Table 1), with a focus on the changes of *P. distasonis* in six types of liver and intestinal diseases, in order to provide clinical references and theoretical support for the application of *P. distasonis*.

**TABLE 1:** Pathways and mechanisms of action of *P. distasonis* in the treatment of diseases.

Strain	Disease	Pathway	Mechanism of action	References
*P. distasonis* CGMCC1.30169	Obesity and metabolic dysfunctions	Bile acid metabolism	*P. distasonis* and its production (UDCA, LCA, and succinate) improved the Claudin‐1 and Zo‐1, increased succinate to activate FBPase, and promoted IGN in jejunum and increased FGF15 in ileum and serum.	^19^
*P. distasonis* PF‐BaE5 and PF‐BaE11	TNBS‐induced colitis	Inflammatory reaction	*P. distasonis* primed dendritic cells to induce regulatory T lymphocytes from naïve CD4 T cells, reduced the expression of proinflammatory factors, increased levels of IL‐10 producing CD4+ FoxP3+ T cells and enhanced expression of the gene encoding Ebi3.	^22^
*P. distasonis* ATCC‐8503	Hepatic fibrosis	Bile acid metabolism	*P. distasonis* increased the gene level of *Oatp4* and *Bsep* to reverse liver BAs in mice with hepatic fibrosis. *P. distasonis* promoted the BSH activity in cecum and reduced the level of TCDCA, which increased in hepatic fibrosis and induced pyroptosis to damage hepatocytes and HSCs.	^66^
*P. distasonis* ATCC 8503	Type 2 diabetes	Inflammatory reaction	*P. distasonis* increased indoleacrylic acid and promoted the secretion of Il22 to maintain the intestinal barrier.	^80^
*P. distasonis* NSP007	Insulin resistance	Nucleotide metabolism	*P. distasonis* and its product nicotinic acid enhanced the integrity of the intestinal barrier, reduced systemic inflammation.	^82^
*P. distasonis* ATCC‐8503	Testicular dysfunction	Polyamine metabolism	*P. distasonis* ameliorated triptolide‐induced testicular dysfunction and increased spermine that upregulate Hsp70s and improved oxidative stress.	^95^
*P. distasonis* ATCC‐8503	Nonalcoholic fatty liver disease	Fatty acid and bile acid metabolism	*P. distasonis*‐derived γ‐linolenic acid enhanced lipid catabolism and changed the BA synthesis pathway in NAFLD.	^99^
*P. distasonis* ATCC 8503	Obesity after calorie restriction	Bile acid metabolism	*P. distasonis* enhanced glucose and energy metabolism, decreased fasting blood glucose levels, improved glucose tolerance and insulin sensitivity, as well as increased thermogenesis, promoted the synthesis of UDCA and LCA to induce the secretion of GLP‐1 and increased the level of UCP1.	^109^
*P. distasonis* BAA‐1295	Arthritis	Bile acid metabolism	*P. distasonis* derived BAs promoted M2 polarization of macrophages, inhibited the differentiation of Th17 cells and reversed the elevation of inflammatory factors, inhibited the differentiation of Th17 cells in vitro through suppressing the expression of RORγt.	^113^
*P. distasonis* DSM 29491	Ulcerative colitis	Immune system	*P. distasonis* promoted intestinal ILC3, enhanced the expression of mucins and claudins proteins, and increased the number of goblet cells.	^116^
*P. distasonis* ATCC 8503	*KRAS* p.G12D‐mutant colorectal cancer	Immune system	*P. distasonis* inhibited the invasion of *F. nucleatum*, prevented it from binding to DHX15, reduced the expression of Ki67 in colonic organoids, inhibited intestinal epithelial over proliferation.	^125^
*P. distasonis* ATCC 8503	Colorectal cancer	Inflammatory reaction	*P. distasonis* decreased the expression of Il‐4, Il‐6, and Tnf‐α, and increased the expression of Il‐10 and Tgf‐β.	^129^
*P. distasonis* DM‐PD18	Alcohol‐induced liver injury	Amino acid metabolism	*P. distasonis‐*mediated regulation of the gut–liver axis ameliorated alcohol‐induced dysbiosis of microbiota metabolites profile, primarily affecting amino acid metabolism and bile acids metabolism.	^140^
*P. distasonis* DSM 108218	Nonalcoholic steatohepatitis	Fatty acid metabolism	*P. distasonis* reduced serum LPS, promoted the expression of tight junction proteins E‐cadherin, restored intestinal barrier function. Additionally, *P. distasonis* metabolized inulin to produce pentadecanoic acid, which has a therapeutic effect on intestinal barrier.	^148^
*P. distasonis* DSM 20,701	Chronic alcohol‐related liver disease	Fatty acid metabolism	*P. distasonis* and *P. distasonis*‐ derived propionic acid activated MUP1 expression in the liver, and significantly reduced macrophage infiltration in mice fed with alcohol.	^156^
*P. distasonis*	Bladder cancer	Immune system	*P. distasonis* augmented the infiltration of CD4T and CD8T cells into the tumor.	^167^
*P. distasonis* F1‐2	Chronic abdominal pain	Inflammatory reaction	*P. distasonis* F1‐2 relieved visceral pain associated with leaky gut by reducing nociceptor response to capsaicin, inflammatory soup, and bradykinin stimulation.	^135^
*P. distasonis ATCC 8503*	Rheumatoid arthritis	Inflammatory reaction	*P. distasonis* used β‐glucosidase to release glycitein from the diet, modulated inflammatory rhythms in rheumatoid arthritis.	^179^

### 4.1 *P*. distasonis and IBD

IBD is an autoimmune inflammatory disease that requires a lifelong management, including ulcerative colitis (UC) and Crohn's disease (CD). Numerous literature reports have documented the presence of gut microbiome imbalances in IBD populations, featured by an enrichment of intestinal germs and a loss in probiotics. Transformation in the constitution and function of intestinal flora directly affect the intestinal environment, which may be a critical factor in the progression of IBD.

Several strains of *P. distasonis* were reported to show immunomodulatory and intestinal barrier strengthening efficacy in vitro and induce the conversion of dendritic cells (DCs) from naive CD4 T cells to regulate T cells and exert anti‐IBD effects in vivo.^22^ In a prospective observational study involving 64 volunteers, it was found that the acute phase protein C reaction protein, LPS‐binding protein, serum amyloid A1, and orosomucoid 1 in overweight/obese IBD patients were significantly lower than ordinary IBD patients, and overweight/obesity could further promote the microbial diversity of UC, as indicated by the increased abundance of some probiotics, including *P. distasonis*, *Alistipes indistinctus*, and *Ruminococcus bromii*.^115^ Therefore, it was speculated that the reduced inflammatory response in IBD patients was related to the increased abundance of probiotics. Mice with obvious resistance to dextran sulfate sodium (DSS)‐induced colitis had significantly increased the abundance of *Akkermansia muciniphila* and *P. distasonis* among the gut microbiota, which were subsequently colonized to demonstrate synergistic improvement of intestinal epithelial integrity and protect against acute colitis.^116^ In addition to live *P. distasonis* alleviating IBD, bacterial components and bacterial by‐products are also able to reduce inflammation. The insoluble membranous fraction isolated from *P. distasonis* (mPd) after centrifugation was fed to mice, which inhibited the expression of proinflammatory factor, prevented the decrease of serum antibodies and gut microecological imbalance induced by DSS.^20^ Colonization of the intestine of SAMP1 mice with *P. distasonis* did not increase intestinal inflammation and other behavioral indicators, but induced depression‐like behaviors.^117^ Some drugs or natural products exert anticolitis effects by regulating intestinal flora. Enteromorpha clathrata polysaccharide enhanced the level of *Parabacteroides spp*. in vivo. Enteromorpha clathrata polysaccharide could promote the development of *P. distasonis* F1‐28 in vitro and increase its generation of SCFAs.^118^

Although many studies suggested the positive character of *P. distasonis* in IBD, different opinions still exist. Pglyrp regulates intestinal flora by enhancing the abundance of *Prevotella falsenii* and *P. distasonis* in the gut. Subsequent single‐bacterium gavage experiments showed that *P. distasonis* aggravated DSS‐induced colitis in mice.^119^ In CD, *P. distasonis* was the most typical growth, while *Faecalibacterium prausnitzii* and *B. fragilis* were obviously lessened.^120^ A total of 80 bacterial strains were isolated from the small intestinal mucosa of CD patients after anaerobic culture, and nine strains were identified on the basis of their link with CD, which included *P. distasonis*.^121^ A strain of *P. distasonis* named CAVFT‐Har46 was isolated from intestinal cavernous fistulous tract (CavFT) microlesions of a CD patient and was tested for its complete genome sequence.^122^ *P. distasonis* has promising applications in the treatment of IBD, but its possible pathogenic effects and interference with the nervous system still need to be vigilant in clinical practice.

### 4.2 *P. distasonis* and colorectal cancer

Colorectal cancer (CRC) is one of the most ordinary and fatal cancer of the alimentary tract. Tumor localization of CRC is in direct contact with trillions of gut microbes. Altered microbial profiles induce dysbiosis and CRC.^123^ The gut microbiota has been shown to be disturbed in the early stages of colorectal neoplasia and aggravated during disease progression. Gut microbiota is critical in HFD‐associated colorectal tumorigenesis.^124^ In germ‐free mice treated with azoxymethane (AOM) under HFD, Alistipessp.Marseille‐P5997 and Alistipessp.5CPEGH6 accumulated in the gut of the CRC model.^124^ *P. distasonis* was depleted, and intestinal barrier function was impaired, indicating that HFD‐modulated gut microbiota promoted the occurrence of colorectal tumors.^124^ Gut microbiota plays a central role in CRC. Many different kinds of microbes are of the relationships of generation and restriction in the gut. *Fusobacterium nucleatus* promoted colorectal neoplasia in *Villin‐Cre/Kras^G12D+/^* ^−^mice, while *P. distasonis* could antagonize it and reduce the number and size of tumors.^125^ Smoke exposure is one of the main inducer of CRC. Significant differences of gut bacteria abundance were monitored in the smoking mice, including the enrichment of *Eggerthella lenta* and loss of *P. distasonis*.^126^ Compared with wild‐type mice, the amount of *Clostridium septicum* in the stool of TGFB signaling deficient mice was increased, while the abundance of *P. distasonis* was decreased, which was strongly associated with colon tumor development.^127^ The depletion of *P. distasonis* was correlated with increased tumor burden.^128^ Loss of *P. distasonis* contributes to CRC progression. Dietary supplementation of *P. distasonis* appears to be a potential therapeutic strategy for CRC. *P. distasonis* diet mice have a lower toll‐like receptor 4 (*Tlr4*), *Il‐4*, and tumor necrosis factor αlpha (*Tnf‐α*) expression in colon and a higher colonic interleukin 10 (*Il‐10*) and transforming growth factor beta (*Tgf‐β*) expression. *P. distasonis* could increase colonic concentration of the proteins Zonula occludens‐1 (Zo‐1) and Occludin.^129^ In addition to directly supplementing live *P. distasonis*, its outer membrane vesicles inhibited the proliferation of CT26 colon cancer cells in vitro and suppressed tumor growth in CT26 tumor‐bearing mice.^130^

Nevertheless, one study reported that an increased abundance of *P. distasonis* was negatively associated with Kupffer cells and positively associated with liver metastasis of CRC after vancomycin supplementation. Here is a lack of direct evidence that *P. distasonis* is the trigger for liver metastasis in CRC. A lot of research and further evidence are needed to clarify the confusion.^131^ In summary, *P. distasonis* is showing a significant protective effect on the progression of CRC.

### 4.3 *P. distasonis* and other bowel diseases

Many factors may induce intestinal injury. *P. distasonis* has also demonstrated a broad spectrum of protective effects against other enteropathies. Gastrointestinal dysfunction is a common complication in diabetic patients. The abnormal proliferation of gram‐negative bacteria in patients with type 2 diabetes resulted in increased intestinal permeability and impaired intestinal barrier by producing large amounts of LPS.^132^ Supplementation of *P. distasonis* ATCC 8503 for 12 weeks decreased the concentration of LPS in serum, inhibited the expression of *Il‐6*, *Tnf‐α*, and *Il‐1β*, stimulated the excretion of IL‐10, and promoted the expression of colon Occludin, Claudin‐1, Claudin‐2, and Zo‐1.^80^ The protective effects of some natural products are closely associated with altered gut microbiota. Poria cocos polysaccharide (PCP) promoted the expression of intestinal tight junction protein Zo‐1 and regulated seven characteristic flora in cecum of antibiotic‐associated diarrhea mice, which involve *P. distasonis*.^133^ After ileocecal excision, the supplementation of tributyrin lessened the concentration of TNF‐α and IL‐6, augmented the abundance of *Bacteroides thetaiotomicorn* and *P. distasonis*, and alleviated colon inflammation.^134^ Some patients with irritable bowel syndrome (IBS) have colonic hypersensitivity, which is characterized by urgent bowel movements, bloating, and abdominal pain. Although the mechanism of chronic visceral hypersensitivity in IBS patients is still unclear, it does not prevent researchers from looking for a solution. *P. distasonis* F1‐2 isolated from a healthy donor has neuroinhibitory properties.^135^ Oral administration of F1‐2 reduced intracolonic pressure and intestinal permeability, significantly improving colon hypersensitivity induced by 0.5% DSS and Citrobacter rodentium postinfectious, respectively.^135^ However, the efficacy of *P. distasonis* on IBS was worth pondering in another study. Transformation in gut microbiota were present in loperamide‐induced constipation and constipation‐dominated IBS featured by the increased *Bacteroides ovatus* and *P. distasonis* in both models.^136^ The causal relationship between *P. distasonis* and the two models is unknown, and a lot of work is still needed to explore in the future.

### 4.4 *P. distasonis* and cholestasis

Bile secretion and excretion disorders lead to excessive accumulation of bile in the liver and systemic circulation, resulting in abnormal liver function and skin status, which called cholestasis. Recently, the changes of *P. distasonis* in cholestasis have been reported. Initially, it was noted that the relative abundance of *P. distasonis* was higher in healthy infants than in cholestatic infants.^137^ Some researchers conducted an initial analysis of data from the BIG Data Center (CRA001920) and found a significant correlation between clinical cholestasis and decreased levels of *Bacteroidetes* and *Parabacteroides*.^66^ The changes in *P. distasonis* were observed in the feces of 13 cholestasis patients and found that its abundance in patients was significantly lower than that of healthy individuals (*n* = 10).^66^ It suggested that the level of *P. distasonis* could be inhibited in cholestasis.

BAs are the main components of bile and regulating the pathways related to BA synthesis and excretion is a method to improve cholestasis. Cholesterol 7 alpha‐hydroxylase (*Cyp7a1*) and cholesterol 27 alpha‐hydroxylase (*Cyp27a1*) are the two pathways that initiate BA synthesis.^138^ The bile salt export pump (*Bsep* ) is the microvilli efflux transporter for BAs, while Na+‐taurocholate cotransporting polypeptide (*Ntcp*) is the basolateral absorptive transporter for BAs.^139^ A recent report indicated that *P. distasonis* inhibited the expression levels of *Cyp7a1* and *Cyp27a1*, promoted the expression of *Bsep* and *Ntcp*, and regulated the synthesis and secretion of BAs in the liver.^140^ Therefore, *P. distasonis* may be a potential approach for clinical cholestasis treatment.

### 4.5 *P. distasonis* and liver fibrosis

Hepatic fibrosis is a phenomenon of abnormal hyperplasia of connective tissue caused by multiple causative factors‐induced liver injury. Long‐term cholestasis may be developed into liver fibrosis, and liver fibrosis may further develop into cirrhosis on a long‐term basis. Patients with hepatic fibrosis in Rio de Janeiro, Brazil, and Spain were reported to reduce *P. distasonis* in the gut.^141^, ^142^ It was also found that the feces of 17 patients with liver fibrosis in Kunming, China, showed a significant decrease in *P. distasonis*.^66^ The abundance of *P. distasonis* was decreased in both senile sepsis related liver injury rats and thioacetamide‐induced liver fibrosis mice.^66^, ^143^ It suggests that *P. distasonis* is negatively correlated with liver fibrosis. BSHs are highly conserved in major classes of intestinal microbial phyla, and they differ among bacteria due to their preference for binding to glycine or taurine‐containing BAs.^144^ Clinical data showed that *P. distasonis* is positively correlated with BSH activity in patients with hepatic fibrosis.^66^ Multiple strains of *P. distasonis* regulated BA metabolism through BSH, including *P. distasonis* DSM 20701, *P. distasonis* CGMCC 1.30169, and *P. distasonis* ATCC 8503.^19^, ^66^, ^104^ Supplementation of *P. distasonis* may be a therapeutic strategy. Transplantation of *P. distasonis* ATCC 8503 improved liver histology in mice with hepatic fibrosis and reduced alanine aminotransferase (ALT) and the gene and protein expression of proinflammatory factors and liver fibrosis genes, including IL‐1β, IL6, TIMP1, and TGF‐β.^66^ In general, *P. distasonis* has an antifibrosis effect in liver.

### 4.6 *P. distasonis* and fatty liver disease

Fatty liver is characterized by hepatocyte steatosis, which is common in nonalcoholic liver disease (NAFLD). *P. distasonis* is associated with the development of fatty liver. Compared with the control group, the abundance of *P. distasonis* in mice with high‐fat‐diet induced hepatic steatosis decreased from 2.28 ± 0.43 to 0.39 ± 0.12%.^145^ Some researchers found that *P. distasonis* fluctuated during the course of the disease, with the highest abundance on the 10th week induced by high‐fat and high‐cholesterol diets. With the development of hyperlipidemia, the abundance of *P. distasonis* was decreased until the 30th day.^146^ A clinical investigation also found that the abundance of *P. distasonis* was significantly higher in fatty liver than in NAFLD.^147^ NAFLD was officially renamed as metabolic dysfunction‐associated fatty liver disease (MAFLD) in 2020 due to unreasonable exclusivism. As MAFLD progresses, it may lead to liver inflammation and eventually develop into nonalcoholic steatohepatitis (NASH). Metagenomic sequencing revealed that *P. distasonis* in the intestines of mice fed a HFD lacking choline for 16 weeks was depleted, while the liver steatosis and necroinflammatory area in mice fed a liquid diet containing *P. distasonis* were significantly lower.^148^ The HFD is one of the triggers of diabetes. Clinical data showed that *P. distasonis* is significantly lower in type 2 diabetic patients (*n* = 14) than in healthy subjects (*n* = 91).^80^ Liver dyslipidemia is also considered to be associated with diabetes. It was found that *P. distasonis* significantly improved insulin resistance (IR) index, lowered triglycerides (TG), total cholesterol (TC), and low‐density lipoprotein (LDL‐C) levels, indicating that it could improve lipid metabolic disorders in T2D rats.^80^ It was also found that transplantation of *P. distasonis* could decrease the serum TC of *ob/ob* mice by 20% and reduced the serum concentrations of LDL‐C, serum free fatty acids, and serum TG, contributing to alleviate hepatic steatosis.^19^ In conclusion, the lipid‐lowering function of *P. distasonis* supports its therapeutic potential in fatty liver disease.

The growing evidences suggested that sarcopenia was associated with IR.^149^ Fat accumulation leads to a condition calling muscle‐wasting obesity, which is common among older adults.^150^ The ultimate outcome of fatty liver disease is cirrhosis, while sarcopenia is one of the frequent complications during advanced liver disease.^149^ Accumulating evidences suggested that gut microbiota was involved in the pathophysiology of musculoskeletal diseases through the gut muscle axis.^151^, ^152^ Gut microbiome imbalance is a major contributing factor to sarcopenia.^153^ A clinical investigation was conducted in 62 patients with metastatic renal cell carcinoma, among which patients 25 had sarcopenia. It was found that *P. distasonis* and *Dialister* species were strongly associated with sarcopenia, and the gluconeogenesis contributed to sarcopenia.^154^ Another study indicated that *P. distasonis* effectively improved the fasted blood glucose, glycated serum protein, and oral glucose tolerance test in T2D rats, indicating that *P. distasonis* has excellent glucose control ability.^80^ These results pointed out a beneficial effect of *P. distasonis* on sarcopenia, and supplementation of *P. distasonis* contributes to alleviate or delay its progression.

Individuals with excessive alcohol use are prone to develop alcoholic liver disease (ALD), and liver steatosis is considered to be the earliest symptom.^155^ Alcohol intake increases the levels of TC and TG in the serum and LDL‐C in the liver, leading to liver fat accumulation.^140^ After 14 weeks of alcohol liquid diet intake, liver fat droplets, inflammation, and macrophage accumulation were observed in the alcohol mice, while the administration of *P. distasonis* led to a significant decrease in the levels of AST, AST, TG, and mRNA levels of endoplasmic reticulum (ER) stress‐related genes in alcohol mice.^156^ *P. distasonis* has great potential to alleviate ALD and make a significant contribution to ALD intervention measures in the future. In term of MAFLD, obesity, diabetes, sarcopenia, and alcohol‐related liver disease, metabolic dysregulation is the common characteristic. Therefore, it is predicted that *P. distasonis* may be a potential therapeutic option for these metabolic syndromes.

**FIGURE 3:**
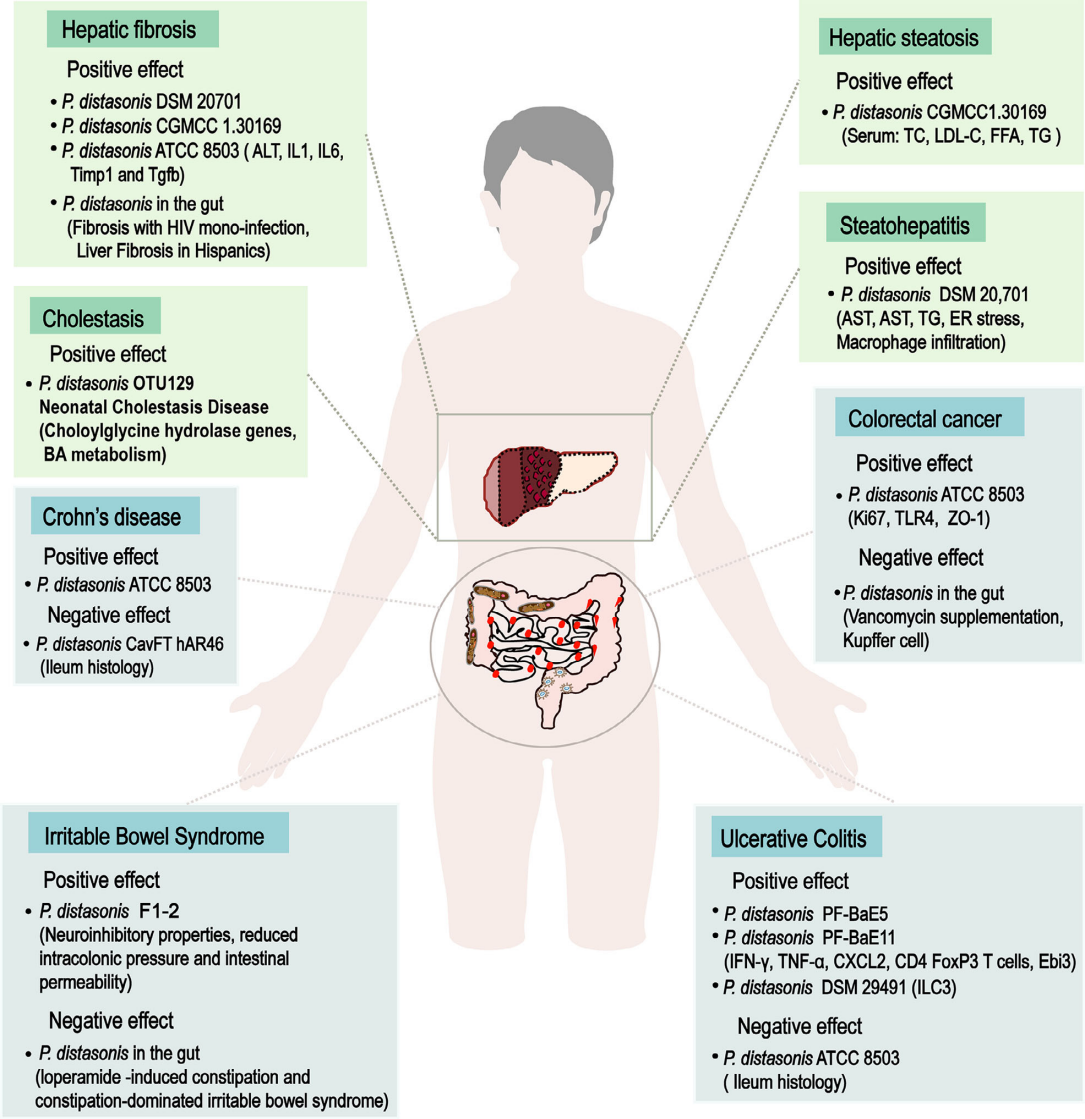
The role of *P. distasonis* in liver disease and intestinal disease. *P. distasonis* presented one‐sided protection in liver disease, while showed a double‐edged sword effect except CRC in intestinal disease.

The abundance changes of *P. distasonis* were summarized in three types of intestinal diseases and three types of liver diseases. It was demonstrated that *P. distasonis* tended to have a protective role in liver diseases, while is dual nature in intestinal diseases (Figure 3). The possible negative effect of *P. distasonis* still needs further study to perform its greater functional value.

## 5 ACTION MECHANISM OF *P. DISTASONIS*

Gut microbiota are able to influence host health through different pathways. Here, we searched the recent reports about the relationship between *P. distasonis* and various diseases. Five mechanisms of action were summarized, including immune regulation, improving intestinal barrier function, interfering with BA receptors, glucose homeostat. A more precise understanding of the interactions between *P. distasonis* and biomolecules may be achieved through these mechanisms research, which is beneficial for drug design and development for *P. distasonis* in the future.

### 5.1 Immunomodulation

Bacteria generally colonize in the gut and direct contact with the mucosal surface of the bowel. The intestinal epithelium was mixed with certain lymphocytes, DCs, microfold cells, and goblet cells. In the lamina propria, there are lymphoid follicles, effector T and B cells, macrophages, and NK cells.

Intestinal immunity is essential for mucosal homeostasis. Receptor retinoic acid‐related orphan receptor gamma t (RORγt) type 3 innate lymphoid cells (ILC3) regulated intestinal homeostasis by producing granulocyte‐macrophage colony‐stimulating factors, which in turn acted on macrophages and DCs.^157^ Mucosal homeostasis provides a habitat for intestinal flora. The gut microbiota supported the development of host metabolism by providing beneficial nutrients (such as BAs and SCFAs), in addition to regulating the maturation of the gut immune population^158^ (such as ILC3,^159^ CD8 T cells,^160^ CD4 T cells,^161^ DC,^162^ T helper 1^163^) by producing signals. *P. distasonis* facilitated the intestinal colonization of *A. muciniphila* and amplified the protective effect of *A. muciniphila* on acute colitis by further promoting intestinal ILC3, enhancing the expression of mucins and claudins proteins, and increasing the number of goblet cells.^116^ When p.G12D was mutated, ERK/STAT3 signal was activated and DHX15 was upregulated. *P. distasonis* inhibited the invasion of *F. nucleatum*, prevented it from binding to DHX15, reduced the expression of Ki67 in colonic organoids, inhibited intestinal epithelial over proliferation, and alleviated the progression of CRC with KRAS p.G12D mutation.^125^

*P. distasonis* stimulates mature immune cells to secrete antibodies, mobilize the systemic immune system and killer system, and eliminate these aging lesions, mutated tissues (such as tumor cells), and exotic pathogenic microorganisms. Initially, some scholars found that *Bacteroides* enhanced its antitumor effect and reduced gastrointestinal toxicity in melanoma patients who received CTLA‐4 blocking therapy.^164^, ^165^ It was further demonstrated that *P. distasonis* enhances anticancer immunity by inducing the production of IFN‐γcd8 T cells.^127^ *P. distasonis* blocked the activation of *Tlr4*, decreased the expression of Il‐4, Il‐6, and Tnf‐α, increased the expression of IL‐10 and TGF‐ β, and inhibited the expression of MyD88 and pAkt in colon of CRC mice.^129^ *P. distasonis* stimulated anti‐inflammatory IL‐10‐expressing human CD4CD25 T cells and IL‐10FoxP3 Tregs in mice.^166^

Bacteria is able to induce the differentiation of immune cells. It was reported that two strains of *P. distasonis* PF‐BaE5 and PF‐BaE11 induced the conversion of DCs from naive CD4 T cells to regulate T cells, resulting in increased levels of IL‐10 producing CD4+ FoxP3+ T cells and enhanced expression of the gene encoding Ebi3.^22^ Supplementation of *P. distasonis* augmented the infiltration of CD4T and CD8T cells into the tumor, significantly upregulated key immune response pathways, including the PI3K/Akt signaling pathway and PPAR signaling pathway, and potentiated the antitumor efficacy of α‐PD‐1 mAb.^167^ *P. distasonis* also has the same significant anti‐inflammatory effect in vitro. The results showed in vitro experiments that *P. distasonis* CS15 and CS17 could stimulate peripheral blood mononuclear cells induced by LPS to increase the production of IL‐1RA and IL‐10.^168^ *P. distasonis* MRx0005 significantly decreased the IL‐6 level of U373 cells induced by LPS.^73^ *P. distasonis* exerts its anti‐inflammatory effect by inhibiting LPS‐induced IL‐8 release from HT‐29 cells.^169^

**FIGURE 4:**
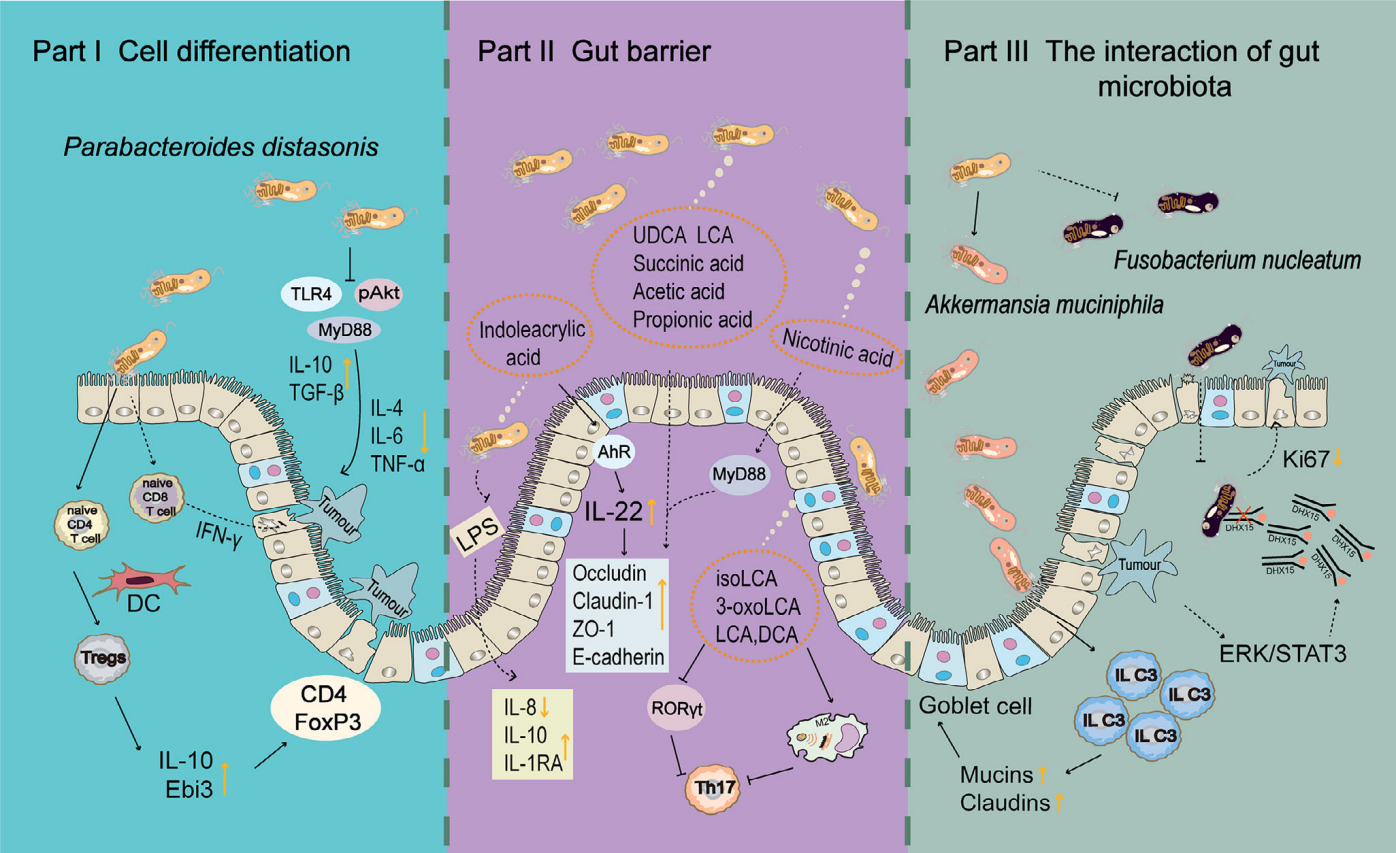
The regulatory effect of *P. distasonis* and its derivatives on host immunity. Part I. *P. distasonis* and its metabolites could promote cell differentiation, transform dendritic cells from naive CD4 T cells to Tregs cells, and promote IL‐10 expression. At the same time, *P. distasonis* could also induce naive CD8 T cells to secrete IFN‐γ and strengthen the defense against tumor cells. Part II. *P. distasonis* and their metabolites could strengthen intestinal barrier function. *P. distasonis* could reduce LPS production, decrease the expression of proinflammatory factor IL‐8, and increase IL‐10 and IL‐1RA. The bile acid, short‐chain fatty acid, indoleacrylic acid, and niacin produced by *P. distasonis* could promote the expression of intestinal tight junction. Part III. *P. distasonis* interacted with other gut microbiota, mainly promoting the colonization of *Akkermansia muciniphila* and inhibiting the invasion of *Fusobacterium nucleatum*. MyD88, myeloid differentiation primary response protein 88; TLR4, Toll‐like receptor 4; pAkt, phosphorylated protein kinase, strain AK, thymoma.

In addition to the direct effect of *P. distasonis*, its derivatives have immunoregulatory effects. *P. distasonis*‐derived BAs promoted M2 polarization of macrophages, which inhibited the differentiation of Th17 cells and reversed the elevation of inflammatory factors in arthritis mice.^113^ 3‐OxoLCA and isoLCA also inhibit the differentiation of Th17 cells in vitro through suppressing the expression of RORγt, while it had no effect on the differentiation of Treg cells.^113^ The mPd prevented the increase of several proinflammatory cytokines (IFN‐γ, IL12, IL‐17, and IL‐6) induced by DSS, increased mPd‐specific serum antibodies IgA and IgG, and stabilized the gut microbial ecology.^20^ In conclusion, *P. distasonis* induces the differentiation of immune cells and secrete antibodies, which has significant anti‐inflammatory effects in vitro and in vivo (Figure 4).

### 5.2 Improves intestinal barrier

The microorganism living in the gut is the main force of intestinal homeostasis, and its change directly affects the intestinal barrier function. The effect may also be positive or negative. For example, *Akkmensia muciniphila* and *Bacillus subtilis* preserve the intestinal barrier, while *Desulfovibrio* and *Salmonella Typhimurium* destroy it. Intestinal inflammation altered intestinal barrier function. C‐X‐C chemokine receptor type 4 (CXCR4) expressed by intestinal plasma cells was considered to be an indicator of gut inflammation in humans.^170^ Metformin downregulated the upregulated CXCR4 gene expression in HFD‐fed mice, reversed the nuclear factor kappa B (NF‐κB) signaling pathway, and positively correlates *Rel* and *Rela* gene expression levels with abundances of *P. distasonis*.^171^ As mentioned earlier, *P. distasonis* is able to regulate immunity. It reduced the expression of proinflammatory factors in colitis mice, and restored colonic tight junction protein expression.^22^ In CRC, *P. distasonis* improved the intestinal epithelial barrier in colon of mice and decrease the level of LPS in plasma.^129^ Interestingly, the membrane proteins of *P. distasonis* also increase transepithelial electrical resistance and the ZO‐1 protein in Caco‐2 cell treated with LPS.^129^

The growth and metabolism of bacteria occur in the intestinal cavity, which has attracted many researchers to focus on the metabolites of *P. distasonis*. The results of animal experiments showed that *P. distasonis* F1‐28 alleviated UC and reduced mucosal damage in DSS‐induced mice.^118^ *P. distasonis* F1‐28 produces acetic acid, propionic acid, and succinic acid in vitro, which have been reported to be beneficial for gut barrier integrity, and contribute another reason to understanding the mechanism of *P. distasonis*.^118^ Recent studies have shown that the action of *P. distasonis* is partially mediated by its active metabolites. Live *P. distasonis* and its production (UDCA, LCA and succinate) improve the Claudin‐1 and Zo‐1 in the ileum of obese mice.^19^ The researchers also found that *P. distasonis* treatment was effective in reversing the characteristics of T2D and alleviated the expression of Occludin, Claudin‐1, and Zo‐1 in SD rats.^80^ As mentioned earlier, *P. distasonis* produces indoleacrylic acid; the serum and fecal samples of experimental animals were detected by targeted liquid chromatography‐tandem mass spectrometry, and the treatment of *P. distasonis* increased indoleacrylic acid. Indoleacrylic acid activates AhR in colon and promotes the secretion of Il22,^80^ which has been reported to maintain the intestinal barrier.^172^ *P. distasonis* and its product NA enhance the integrity of the intestinal barrier by activating G‐protein‐coupled receptor 109a in intestine, thereby improving IR, and reducing systemic inflammation in mice.^82^

It was found that *P. distasonis* DSM 108218 could reduce serum LPS, promote the expression of tight junction proteins E‐cadherin, restore intestinal barrier function to suppress NASH, and inhibit hepatic proinflammatory cytokine and chemokine signaling. Additionally, *P. distasonis* is also able to metabolize inulin to produce pentadecanoic acid, which has a therapeutic effect on intestinal barrier. These discovery also indicated that *P. distasonis* was able to metabolize dietary fiber to connect its effects on diseases.^148^ In summary, *P. distasonis* and its metabolites are beneficial for intestinal barrier maintenance.

### 5.3 Interacts with BA receptors

The liver synthesizes BAs from cholesterol and releases them into the intestine, where enzymes produced by microbiota convert primary BAs into secondary BAs, such as DCA and LCA. Disturbances in the BA profiles, especially changes of secondary BAs, are associated with various diseases. The modulatory effects of BAs on metabolic homeostasis primarily occur through their combination with various intracellular nuclear receptors, such as farnesoid X receptor (FXR) and cell surface G protein‐coupled receptors 5 (TGR5).

In one study, *P. distasonis* was the most nodal species in the differential gut flora species‐BA network, which may play a role in the acute aggravation of chronic liver failure by reestablishing intestinal homeostasis through the production of more secondary BAs.^173^ Chaihu‐Shugan‐San (CSS) was reported to increase circulating levels of hyocholic acid and 7‐ketodeoxycholic acid in blood and alter the expression of hippocampal genes involved in BA transport (*Bsep* and *Fxr*) and neurotrophic signaling.^174^ Fecal transplanting experiments showed that the efficacy of CSS was associated with increased intestinal *P. distasonis* abundance.^174^ As mentioned earlier, *P. distasonis* has genes encoding the synthesis of multiple secondary BAs. It has been reported that treatment with *P. distasonis* was able to modify the BA profiles of mice. Increased LCA and UDCA reduce hyperlipidemia by activating the FXR pathway, and increase fibroblast growth factor 15 (Fgf15) in ileum and serum to restore gut barrier integrity.^19^ γ‐Linolenic acid, a fatty acid metabolite of *P. distasonis* ATCC‐8503, enhances lipid catabolism, promotes hepatic FXR, and inhibits the expression of Cyp7a1 in the liver through peroxisome proliferator‐activated receptor alpha (*Pparα*) signaling pathway, which changes the BA synthesis pathway in NAFLD.^99^ *P. distasonis* increased the mRNA level of *Oatp4* and *Bsep*, which may contribute to reverse liver BAs in mice with hepatic fibrosis. *P. distasonis* promoted the BSH activity in cecum and reduce the level of TCDCA, which was increased in hepatic fibrosis and induced pyroptosis to damage hepatocytes and HSCs. In addition, *P. distasonis* inhibited ileal Fxr and decreased the expression of Fxr and organic solute transporter belta protein, which reduce the reabsorption and promote the excretion of BAs.^66^

**FIGURE 5:**
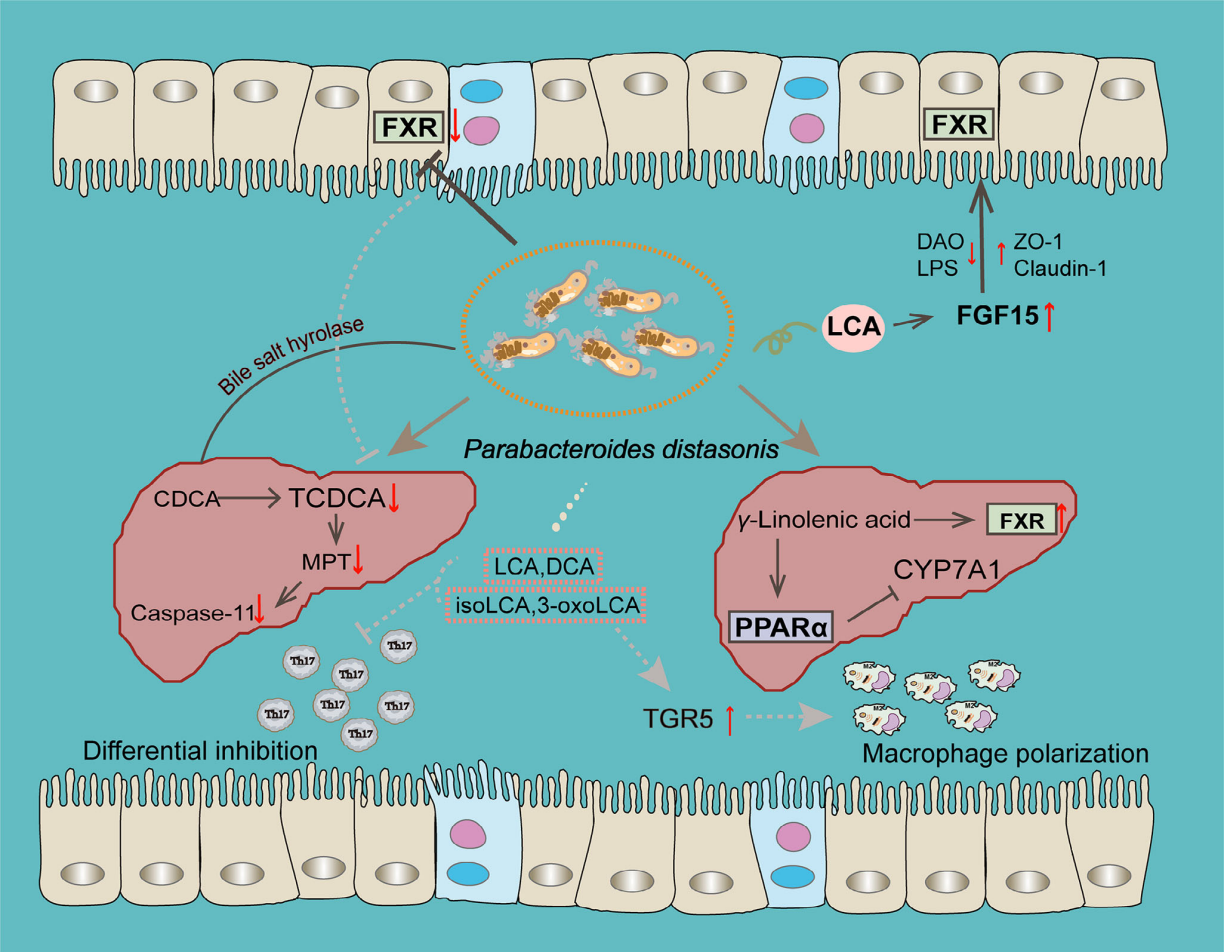
Regulation of bile acid receptors by *P. distasonis* and its derivatives. There are two independent regulatory pathways of *P. distasonis* on liver. First, *P. distasonis* consumed TCDCA concentration through BSH enzyme, thereby alleviating Caspase‐11‐induced hepatocyte apoptosis. Second, γ‐linoleic acid derived from *P. distasonis* activated liver FXR and PPARα at the same time, inhibited the regulation of liver CYP7A1. Three regulatory pathways of *P. distasonis* on intestinal tract were observed. First, the metabolites of *P. distasonis* (LCA, DCA, isoLCA, and 3‐oxoLCA) directly inhibited Th17 cell differentiation. Second, 3‐oxoLCA and isoLCA activated TGR5 and promoted macrophage M2 polarization. Third, the metabolites of *P. distasonis*, LCA, activated FGF15, activated intestinal FXR, and strengthened intestinal barrier function. LCA, lithocholic acid; TGR5, Takeda G protein‐coupled receptor 5; FGF15, fibroblast growth factor.

Recently, researchers have provided solid evidence that *P. distasonis* could generate LCA, DCA, isoLCA, and 3‐oxoLCA.^113^ LCA and DCA are the known ligands of TGR5. Molecular docking and molecular dynamics simulations revealed that isoLCA and 3‐oxoLCA are also the potential ligands for TGR5. It was observed that LCA, DCA, isoLCA, and 3‐oxoLCA reversed the inhibitory effect of LPS on the TGR5 in macrophages and required TGR5 to mediate M2 macrophages polarization, which contribute to alleviating inflammatory arthritis.^113^ Overall, these reports highlight the potential role of *P. distasonis* in host health closely related to BA receptors (Figure 5).

### 5.4 Maintains glucose homeostasis

Glucose meets the energy needs of vital organs and maintains personal health. Various pathways regulate glucose metabolism, including glycogenesis, glycogenolysis, glycolysis, and gluconeogenesis (IGN). Interfering with one of these processes was able to affect the normal metabolism of glucose. *P. distasonis* is linked with the glucose modulatory effects by metformin.^171^ Two *P. distasonis* strains were isolated from human feces, which induce the secretion of the glucagon‐like peptide 1 (GLP‐1) in STC‐1 cells and improve glucose homeostasis in obese mice by reducing plasmatic leptin and increasing the insulin receptor substrate 2.^175^ The regulation of *P. distasonis* on host metabolism is not only itself, but also closely related to its metabolites. A previous report also confirmed that *P. distasonis* activated intestinal IGN and reduced obesity and metabolic dysfunction. *P. distasonis* increases succinic acid to activate fructose‐1, 6‐ bisphosphatase (FBPase) and promote IGN in jejunum.^19^ The administration of *P. distasonis* resulted in enhanced glucose and energy metabolism, decreased fasting blood glucose levels, improved glucose tolerance and insulin sensitivity, as well as increased thermogenesis to facilitate weight gain following a period of fasting.^109^ Live *P. distasonis* promotes the synthesis of UDCA and LCA to induce the secretion of GLP‐1 and increases the level of uncoupling protein 1 (UCP1), which increase glucose homeostasis and energy consumption.^109^ In conclusion, *P. distasonis* has a regulatory effect on glucose metabolism.

### 5.5 Other potential mechanisms

The metabolic regulation of *P. distasonis* is mainly shown in two aspects. One is to regulate host metabolism, and the other is to regulate the secreted metabolites. Depletion of *P. distasonis* causes changes in urea cycle, l‐citrulline biosynthesis, and creatinine degradation pathway, as well as B vitamins and lipoic acid synthesis in patients with hepatic fibrosis in Spain.^142^ *P. distasonis* is also closely related to tryptophan metabolism. Single bacterial colonization experiments have demonstrated that *P. distasonis* induced the decrease of indole‐formaldehyde and the increase of indole‐3‐lactic acid, and this metabolic imbalance directly regulates depression‐like behavior.^79^ Mice with psoriatic skin phenotype showed an intestinal microbiome imbalance, that is, the increase of *Prevotella* and decrease of *P. distasonis*. With the recovery of gut flora, host metabolism in mice was regulated, and the reduction of oleic acid and stearic acid reduces Th17 and monocyte‐derived DCs infiltration in the skin lesion area in vivo, as well as reduced the secretion of IL‐23 by avoiding the stimulation of DCs in vitro.^176^ A recent study was the first to provide positive evidence suggesting that *P. distasonis* has the potential to enhance propionic acid production. Specifically, propionic acid derived from *P. distasonis* activated MUP1 expression in the liver, improved ER stress, and significantly reduced macrophage infiltration in mice fed with alcohol.^156^ Spermine is one of the most prevalent and important polyamines, and has wide functions such as antioxidation, inhibition of lipid synthesis, anabolism, maintenance of intestinal homeostasis, and promotion of immune system maturation.^177^ *P. distasonis* ameliorated triptolide‐induced testicular dysfunction and increased spermine that upregulate heat shock protein 70s (Hsp70s) and improve oxidative stress.^95^ The brain–gut axis has received increasing attention. The microbe released signals that triggered inflammatory mediators to act directly on the nervous system, producing pain signals that traveled to the periphery.^178^ *P. distasonis* F1‐2 relieved visceral pain associated with leaky gut by reducing nociceptor response to capsaicin, inflammatory soup, and bradykinin stimulation.^135^ These works suggest that *P. distasonis* has an independent mechanism in different diseases. Much more research is needed to try to more fully understand the complex relationship between *P. distasonis* and human health.

In conclusion, these action mechanisms provide the support for the potential therapeutic value of *P. distasonis*. A further investigation about these mechanisms will clarify the effective metabolites of *P. distasonis* and administration formulation to achieve the enhanced therapeutic efficacy and minimized side effects.

## 6 MANIPULATING *P. DISTASONIS* TO PROMOTE BODY HEALTH

The crosstalk between gut microbiota and natural components is a complicated but captivating field and attracts growing attention. On the one hand, as an integral part of the human body, gut microbiota plays the crucial roles in bioavailability and bioactivity of natural components and their biotransformation process. On the other hand, natural components were able to modulate gut microbiota, affecting the composition and its metabolites, signal transduction, or genetic expression. Numerous studies provide evidence of the linkage between pharmacological efficacy of natural components and the structural shift and metabolic activity of gut microbiota, but only few investigate certain species. Natural components especially prebiotics from food sources, as well as various traditional Chinese medicines (TCMs) and some natural products are well known for improving host metabolism and immune system via nourishing gut microbes.^180^, ^181^ Here, we summarize the literatures on the role of natural components regulating the specific gut bacteria *P. distasonis* to provide scientific support for potential novel prevention or therapeutic strategies for gastrointestinal diseases.

### 6.1 Dietary fibers

Dietary fibers mainly include plant carbohydrates that are not recovered by alcohol precipitation, nonstarch polysaccharides, lignin, chitin, pectin, and resistant starch (RS)^182^ and are the most widely studied plant‐derived natural components that modulating gut microbiota.

#### 6.1.1 Plant carbohydrates

Certain natural fibers such as inulin, oligosaccharides, and fructans act as prebiotics. Chicory inulin supplement is currently the most widely used and studied prebiotic. In a study, the prebiotic activity of inulin was tested in an in vitro model of human colon. For the first time, it was reported mainly used by the *Parabacteroides* genus, selectively leading to a significant increase of *P. distasonis*.^183^ Recently, it was reported inulin protects NASH development by enriching potential beneficial bacteria *P. distasonis*, which was depleted in choline‐deficient high‐fat diet‐induced mice NASH model. The underlying mechanism is the inulin‐derived *P. distasonis* and its metabolite pentadecanoic acid alleviated NASH by improving gut barrier and suppressing inflammation.^148^

#### 6.1.2 Nonstarch polysaccharides

Non‐starch polysaccharides (NSPs) are one kind of dietary fiber commonly found in many plants and include a variety of different types of polysaccharides, such as cellulose, pectins, gums, and glucans. Numerous studies have shown many plant polysaccharides are able to repair colon injury and improve gastrointestinal disease by modifying gut bacteria. The fermentation of Chlorella pyrenoidosa polysaccharides (CPP) altered the structure of fecal microbiota in vitro, remarkably decreasing the harmful bacteria and increasing the probiotic bacteria. CPP intervention increased the abundance of *P. distasonis* and SCFAs production, such as acetic acid, propionic acid, and n‐butyric acid. Thus, it may contribute to promote gut health and prevent diseases.^184^ The effect of edible brown algae, containing fermentable dietary fiber polysaccharides alginate and laminaran on rat cecal microbiota was investigated. The result suggested that these two dietary fibers were able to change the microbial composition of cecum in rat and affect the gut microenvironment. Only laminaran increased the abundance of *P. distasonis*, and this difference may be related to the ability of *P. distasonis* to utilize polysaccharides.^185^ A study revealed gut microbiota communities from different donors utilized the sulfated polysaccharides of sea cucumber Stichopus japonicas (SCSPsj) to different degrees in vitro fermentation, demonstrating that SCSPsj benefits the host health by effectively modulating gut microbiota composition. Those gut microbiota communities with *P. distasonis* enriched showed the strongest capability to utilize SCSPsj.^186^ SCSPsj is able to be used by *P. distasonis* to produce small molecules such as organic acids and lipids. In addition, those molecules were significantly consumed by bacteroidales members *Bacteroidales stercoris*, *Bacteroidales vulgatus*, and *Parabacteroides johnsonii*, consequently, promoted the growth of these species through cross‐feeding.^187^ Rice bran has been shown the suppressive effect for colitis and colon cancer in multiple animal models via modifying human gut microbiota. Mice transplanted with rice bran‐modified microbiota displayed improved CRC and distinctly depleted *P. distasonis* is correlated with increased tumor burden.^128^

#### 6.1.3 Resistant dextrins and resistant starch

Resistant dextrins (RD) and resistant starch (RS) are not digested by human enzymes in the small intestine but are fermented in the colon by intestinal bacteria, contributing to produce SCFAs, which have health benefits. Diet supplement with RD NUTRIOSE, a soluble fiber, did not alter the overall composition of human gut microbiota but dramatically increased the abundance of bacterium *P. distasonis*. The response to RD depends on the gene susCD, a starch‐binding membrane protein complex, carried in *P. distasonis* strains.^188^ Interestingly, the structures of RD and RS play an important role in microbiota regulating. For example, a study compared different effects of two RS, high‐amylose maize starch (HAMS) and butyrylated HAMS (HAMSB), on fecal microbiota composition of rats treated with carcinogen AOM. HAMS was found only increased concentration of propionate and developed *R. bromii*‐like bacteria in rats, whereas HAMSB, a chemically modified RS, increased both the propionate and butyrate levels, and was related to the appearance of two nonbutyrate‐producing bacteria, *P. distasonis* and *Lactobacillus gasseri*. Additionally, *P. distasonis*‐related phenotypes were only detected in butyrylated RS group, suggesting that these bacteria may be adaptable and able to deacylate and utilize the starch backbone for growth.^189^ According to a clinical trial, ingestion of HAMSB has the potential to improve human gut health probably because it effectively released esterified butyrate to the gastrointestinal tract, and therefore caused significant change in gut microbial diversity. The release was likely facilitated primarily by *P. distasonis*, of which the increased abundance is positively correlated to HAMSB but not HAMS.^190^

### 6.2 Natural products

Many types of natural compounds such as saponins, flavonoids, alkaloids, and polysaccharides have been proven to possess beneficial effects for intestinal health. Among them, dietary polyphenols have attracted the most attention. However, their extremely low bioavailability limited the mechanistic understanding of their function. Recent research revealed their effects were closely related to the gut microbiota and therefore drove the breakthrough strategy of targeting gut microbiota for gastrointestinal disease by natural products. Polyphenols exert various biological activities such as antioxidation, anti‐inflammation, and antiobesity by adjusting gut microbiota composition.^191^ Liu et al. performed a comparative study of green tea phenolics (GTP) and oxidized tea phenolics (OTP) on antiobesity and their gut microbiota modulating effects. The supplementation of GTP or OTP both restored *P. distasonis* reduction caused by HFD, and GTP was demonstrated better effect.^192^ Compared the fecal samples of patients with or without precancerous lesions, the anaerobic bacteroides *P. distasonis* and equol were only detected in patients without precancerous lesions. The presence of *P. distasonis* in the colon could contribute to the metabolism of soy.^193^ The pomegranate fruit pulp polyphenols (PFP) exhibited significant antiobesity effect in mice fed a HFD. Remarkably, the increased abundance of *P. distasonis* by PFP supplement was negatively correlated with clinical indicators of obesity such as body weights, ALT, TC.^194^ Propolis is another natural product of which the main bioactive substances are polyphenols such as flavonoids. Ethanol extract of propolis (EEP) significantly increased the abundance of *P. distasonis* in HFD‐fed mice to a much higher level than that in Chow mice, which was correlated with the improvement of inflammation, IR, obesity, glucose tolerance, and lipid profile.^191^

The potential protecting effect of Scytosiphon lomentaria fucoidan (SLF) on ALD was assessed in a study. It was demonstrated that SLF reduced the levels of ALT, AST, TG, and TC in ALD mice and its benefits was partly attributed to *P. distasonis* regulating gut–liver axis. SLF increased alcohol intake and reduced levels of Bacteroides and Parabacteroides, particularly *P. distasonis*. *P. distasonis* administration alleviated liver inflammation by suppressing the NF‐кB/MAPK pathway and mitigated liver oxidative stress by activating Nrf2 pathway. The underlying mechanism is closely associated with modulation of gut microbe composition and improved profiles of microbiota metabolites, especially amino acid and BA metabolism.^140^ Lycium barbarum fruit is a widely used food supplement that is rich in plant polysaccharides. Lycium barbarum glycopeptide (LbGP) is a glycopeptide isolated and purified from crude lycium barbarum polysaccharide, which significantly alleviated acute colitis by inhibiting the colonization of harmful bacteria *P. distasonis*. It dominated the intestinal bacterial community during the acute phase of colitis.^195^ Oregano essential oils (OEO) improved rumen digestive ability of cattle. *P. distasonis* was identified among the top five significantly increased species after OEO administration. OEO diets changed rumen content metabolites in beef cattle, and a positive correlation was found between *P. distasonis* and β‐glucosidase, cellulase, propionate, and valerate.^196^

### 6.3 TCMs formulas and active ingredients of TCMs

TCMs such as Si Miao formula, Shenling Baizhu Powder, and Gegen Qinlian Decoction has been used for centuries to treat various gastrointestinal disorders. These TCM formulas and active ingredients of them have been demonstrated exerted therapeutic effects by directly or indirectly interacting with gut microbiota.

The protective effects of *P. distasonis* on metabolic syndrome, such as obesity, diabetes mellitus and NAFLD, have been long and widely investigated. It was reported that Rhizoma Coptidis (RC) alkaloids, including berberine, coptisine, palmatine, and epiberberine, obviously suppressed the richness of *P. distasonis* and alleviated hyperlipidemia. Respectively, the inhibition rates of berberine, coptisine, palmatine, and the total Rhizoma Coptidis alkaloids (TRCA) are 33.3, 47.6, 47.3, and 61.9%.^146^ In another study, RC classically paired with Dolomiaea souliei (VR) was used for gastrointestinal diseases treatment. RC alkaloids gavage decreased gut microbiota diversity, whereas combination of VR and RC reversed the perturbation. However, both RC and VR alone promoted the proportion of *P. distasonis*.^197^

PCP demonstrated the potential prebiotic and therapeutic efficacy in the treatment of antibiotic associated diarrhea in mice via regulating intestinal mucosal barrier and the homeostasis of gut microbiota, specifically restoring seven characteristic species of intestinal flora, including *P. distasonis*.^133^

The commercial herb product *Panax notoginseng saponins* (PNS) includes ginsenosides Rb1, Rd, Re, Rf, Rg1, and notoginsenoside R1 directly shifted the gut microbial community in HFD‐fed obesity mice prior to body weight change, particularly increased the level of *P. distasonis* within a short time. The crosstalk of microbiota and fat via leptin‐AMPK/STAT3 signaling pathway plays an essential role on the antiobesity effects of PNS.^198^ Celastrol from the roots of *Tripterygium Wilfordii* was reported possessing the antiobesity effect and ameliorated hepatic fibrosis via inhibition of intestinal Fxr signaling and decreased TCDCA in liver by promoting the growth of *P. distasonis*.^66^

Natural components have been long widely used in human daily life and clinical practice, and have been proven to possess benefits for maintaining intestinal homeostasis and symbiosis. Recent research revealed that the specific strain *P. distasonis* was involved in the regulatory effects of many natural components on host health (Table 2). Natural components addressed gut dysbiosis by restored the abundance of *P. distasonis*, which contributed to the diversity of microbial composition. Also, it produced metabolites with different physiological functions, such as SCFAs, amino acids, BAs, and lipids. Meanwhile, *P. distasonis* enriched gut microbiota communities could better utilize and transform natural components, making them more easily absorbed and harnessed by the host. This interaction is inseparable for host health, but molecular mechanism studies are few at present, and further exploration is needed.

**TABLE 2:** Natural components regulate *P. distasonis* as a potential therapeutic option for disease.

Natural components type	Name	Gut bacterial alteration	Potential effects and possible mechanism	References
Dietary fiber	Inulin	*P. distasonis*↑	*P. distasonis* and its metabolite pentadecanoic acid alleviated NASH by restoring gut barrier and inhibiting proinflammatory signaling.	^148^
Natural products	SLF	*P. distasonis* ↑	*P. distasonis* alleviated liver inflammation by suppressing the NF‐кB/MAPK pathway and mitigated liver oxidative stress by activating Nrf2 pathway in ALD mice.	^140^
	EEP	*P. distasonis* ↑	*P. distasonis* was positively correlated with improvement of proinflammatory mediators, insulin resistance, obesity, glucose tolerance, and lipid profile.	^191^
	PFP	*P. distasonis* ↑	*P. distasonis* was negatively correlated with indicators of obesity such as body weights, glucose, ALT, AST, TG, TC, LDL‐C, NEFA, and insulin.	^194^
	LbGP	*P. distasonis* ↓	LbGP inhibited the colonization of *P. distasonis* which dominated in acute colitis.	^195^
TCM formulas and active ingredients	Celastrol	*P. distasonis* ↑	*P. distasonis* possessed the antiobesity effect and ameliorated hepatic fibrosis via inhibiting intestinal Fxr signaling and decreased TCDCA in liver.	^66^
	PNS	*P. distasonis* ↓	The crosstalk of *P. distasonis* and fat was through leptin–AMPK/STAT3 signaling pathway.	^198^

## 7 CURRENT RESEARCH IN CLINICAL TRIALS

Regulating dysbiotic intestinal microbiota is a novel strategy for treating diseases, especially those that are difficult to be cured by conventional therapies.^199^, ^200^ Fecal microbiota transplantation (FMT), prebiotics, probiotics, synbiotics, and dietary intervention possess the potential to become measures for treating or preventing diseases.^201^, ^202^ In addition, it may also mitigate adverse effects in patients, improve their quality of life, and support disease treatment.^203^ At present, these methods have been exhibited therapeutic effects in the treatment of *Clostridium difficile* infection,^204^, ^205^ UC,^206^ NAFLD,^207^ and IBS.^208^

In current clinical trials, prebiotics and dietary intervention alter *P. distasonis* and exert modulatory effect. The clinical trials related to *P. distasonis* are summarized in Table 3. Continuous supplementation of HAMSB at a dose of 40 g/day for 28 days resulted in the most significant increase of *P. distasonis* in feces, as well as elevated levels of IL‐10 and TNF‐α in plasma samples, which may contribute to the ability to resist infections.^209^ A high intake of red meat enriched diet is a risk factor for CRC due to its stimulation of a toxic substance O(6)‐methyl‐2‐deoxyguanosine (O(6)MeG). In a clinical trial involving 23 healthy volunteers, the HAMSB diet significantly increased the abundance of *P. distasonis* and the level of stool SCFAs, thus reducing O(6)MeG adducts in rectum.^210^ Inulin increased the abundance of *P. distasonis* and could be transformed by *P. distasonis* in animal experiments.^148^ Consistent with the previous results, supplementation with inulin resulted in an increase in *P. distasonis* in aged individuals. However, the content of SCFAs in feces did not show significant changes in this trial.^211^

**TABLE 3:** Current research of *P. distasonis* in clinical trials.

Method	Numbers of subjects	Main effects	References
HAMSB	Male (*n* = 23) Female (*n* = 18)	HAMSB increased *P. distasonis* and *F. prausnitzii* in feces. HAMSB elevated the levels of IL‐10 and TNF‐α in plasma.	^209^
HAMSB	23 healthy volunteers	HAMSB increased *P. distasonis* in feces. HAMSB diet resisted the increase of O(6)MeG adducts caused by red meat.	^210^
Inulin	24 healthy volunteers	Inulin increased the abundance of *P. distasonis*.	^211^
Intermittent fasting	Male (*n* = 33) Female (*n* = 39)	Intermittent fasting decrease weight, BMI, and atherosclerosis index. *P. distasonis* and *B. thetaiotaomicron* increased in feces.	^212^
Intermittent energy restriction	25 obese volunteers	Intermittent energy restriction reduced weight and TC, ALT, AST, high‐density lipoprotein, and low‐density lipoprotein in serum. Intermittent energy restriction reduced *Escherichia coli* and increased *P. distasonis*.	^213^

Obesity is a risk factor for fatty liver, hepatic fibrosis, and IBD. Intermittent fasting is an effective strategy for weight loss. After fasting, both normal and obese participants were able to reduce weight and body mass index (BMI), accompanied by a significant increase in *P. distasonis*.^212^ In another clinical trial, intermittent energy restriction also increased *P. distasonis* and reduced obesity‐related parameters.^213^ The relationship and ameliorative effects of *P. distasonis* on obesity have been demonstrated in animal studies.^19^, ^175^ Alterations in gut microbiota caused by dietary interventions may contribute to these improvements.

Previous animal studies have shown the role of *P. distasonis* in gastrointestinal and hepatic disease. The effects of dietary intervention and prebiotics on *P. distasonis* have also been well validated in clinical trials. However, the direct role of *P. distasonis* in clinical disease has not been verified yet, more research is required to determine the safety and efficacy of *P. distasonis*.

## 8 CONCLUSIONS AND PERSPECTIVES

*P. distasonis* is one of the popularly studied and potential next‐generation probiotics candidates due to its properties of anti‐inflammation, antiobesity, and gut barrier protection,^21^ which has shown protective effects against various gastrointestinal and hepatic diseases. It should be noted that the mechanisms of *P. distasonis* and its impact on diseases are not well understood, and several aspects require further research. *P. distasonis* exhibits dual effects in some diseases. It displays both protective and damaging effects in animal models of IBD.^22^, ^119^ The variability in outcomes may be caused by differences in strains, modeling methods, and experiments conditions. The role of *P. distasonis* requires further validation in multiple models to draw more definitive conclusions. It is also important to consider the variations among strains from different sources. For instance, *P. distasonis* isolated from healthy human feces demonstrated different abilities to exert anti‐inflammatory effects and protect against intestinal barrier.^22^ Additionally, the invasion and antibiotic resistance are also related to strain differences. To ensure safe use of *P. distasonis*, selecting effective and safe strains for trials or employing genetic manipulation is necessary.^214^

The impact of gut microbiota on active metabolites is currently a widely studied mechanism of action. *P. distasonis* exerts its effects by influencing the production of active metabolites, such as produce indoleacrylic acid that is able to protect intestinal barrier.^80^ In other experiments, components of *P. distasonis* such as its membranous fraction and outer membrane vesicles regulate immune system.^20^, ^130^ These studies indicate that the role of *P. distasonis* is linked to its own structure, metabolites, and secreted products. The proteins and metabolites of bacteria as potential postbiotics could avoid the side effects and uncertainties of bacterial transplantation.^215^ The unique metabolic enzymes of bacteria also demonstrate the therapeutic value in degrading harmful substances to the host and protecting against diseases.^216^ Therefore, utilizing isotope labeling, genetic engineering, high‐throughput sequencing, and other technologies are able to enhance our understanding of the regulatory mechanism of *P. distasonis* and facilitate the development of reliable alternatives.

Natural components with thousands of years enriched clinical experience is a rich source of prebiotics and were proven to possess advantages and effectiveness by research evidence and modern clinical practice. Therefore, in addition to FMT and live biotherapeutic products, targeting *P. distasonis* by natural components provides a promising microbiome‐based intervention strategy or therapeutic option for intestinal and liver disease. To standardize treatment protocols and robust therapeutic effects, better understanding of the mechanism how natural components interact with *P. distasonis* on host health is essential. More scientific attention and research efforts should be paid to these future perspectives. First, natural products, whether extracts or single phytochemicals, regulate gut microbiota by targeting multiple strains, but may not specifically *P. distasonis*, implying potential cross interactions between the microbial communities that require further mechanistic investigation. Second, the different structural characteristics of natural compounds also lead to variations in impacting microbial strains.^217^ It may be possible to selectively enrich or downregulate certain species such as *P. distasonis* for better disease intervention. In addition, despite prospects of natural components, some concerns are innegligible. For example, algal polysaccharides including carrageenan, agar, fucoidan, and ulvan are reported to increase H_2_S, which could affect colonic mitochondrial function. Meanwhile, their thickening/emulsifying properties are also emerged concerns.^218^ Therefore, either using natural components as prebiotic or *P. distasonis* to treat gastrointestinal and hepatic disease requires more clinical trials to confirm.

*P. distasonis* has shown potential in the protection of gastrointestinal and hepatic disease. The complexity of the intestinal environment and the diversity of bacterial functions present challenges for its research. Thoroughly studying its mechanism of action and regulatory methods will contribute to a better understanding of how *P. distasonis* is able to be effectively utilized for health benefits while ensuring its safety in clinical application.

## AUTHOR CONTRIBUTIONS

Jingyi Duan, Qinmei Li, and Yan Cheng wrote and revised the manuscript. Hongning Liu and Weifeng Zhu supervised the manuscript and edit the writing. Fei Li conceived the framework of the article, edited the writing, and provided the funding. All authors read and approved the manuscript.

## CONFLICT OF INTEREST STATEMENT

The authors declare no conflicts of interest.

## ETHICS STATEMENT

Not applicable.

## Data Availability

Not applicable.

## References

[1] Peery AF , Crockett SD , Murphy CC , et al. Burden and cost of gastrointestinal, liver, and pancreatic diseases in the United States: Update 2021. Gastroenterology. 2022;162(2):621‐644. 34678215 10.1053/j.gastro.2021.10.017 PMC10756322

[2] Seyed Tabib NS, Madgwick M , Sudhakar P , Verstockt B , Korcsmaros T , Vermeire S . Big data in IBD: big progress for clinical practice. Gut. 2020;69(8):1520‐1532. 32111636 10.1136/gutjnl-2019-320065 PMC7398484

[3] Pang Y , Lv J , Kartsonaki C , et al. Metabolic risk factors, genetic predisposition, and risk of severe liver disease in Chinese: a prospective study of 0.5 million people. Am J Clin Nutr. 2021;114(2):496‐504. 33964851 10.1093/ajcn/nqab099

[4] Camilleri M , Malhi H , Acosta A . Gastrointestinal complications of obesity. Gastroenterology. 2017;152(7):1656‐1670. 28192107 10.1053/j.gastro.2016.12.052 PMC5609829

[5] Corsello A , Pugliese D , Gasbarrini A , Armuzzi A . Diet and nutrients in gastrointestinal chronic diseases. Nutrients. 2020;12(9):2693. 32899273 10.3390/nu12092693 PMC7551310

[6] Wu K , Luo Q , Liu Y , Li A , Xia D , Sun X . Causal relationship between gut microbiota and gastrointestinal diseases: a mendelian randomization study. J Transl Med. 2024;22(1):92. 38263233 10.1186/s12967-024-04894-5 PMC10804519

[7] White LS , Van den Bogaerde J , Kamm M . The gut microbiota: cause and cure of gut diseases. Med J Aust. 2018;209(7):312‐317. 30257633 10.5694/mja17.01067

[8] Boursier J , Mueller O , Barret M , et al. The severity of nonalcoholic fatty liver disease is associated with gut dysbiosis and shift in the metabolic function of the gut microbiota. Hepatology. 2016;63(3):764‐775. 26600078 10.1002/hep.28356 PMC4975935

[9] Franzosa EA , Sirota‐Madi A , Avila‐Pacheco J , et al. Gut microbiome structure and metabolic activity in inflammatory bowel disease. Nat Microbiol. 2019;4(2):293‐305. 30531976 10.1038/s41564-018-0306-4 PMC6342642

[10] O'Hara AM , Shanahan F . The gut flora as a forgotten organ. Embo Rep. 2006;7(7):688‐693. 16819463 10.1038/sj.embor.7400731 PMC1500832

[11] Valdes AM , Walter J , Segal E , Spector TD . Role of the gut microbiota in nutrition and health. BMJ. 2018;361:k2179. 29899036 10.1136/bmj.k2179 PMC6000740

[12] Kelly CJ , Zheng L , Campbell EL , et al. Crosstalk between microbiota‐derived short‐chain fatty acids and intestinal epithelial HIF augments tissue barrier function. Cell Host Microbe. 2015;17(5):662‐671. 25865369 10.1016/j.chom.2015.03.005 PMC4433427

[13] Desai MS , Seekatz AM , Koropatkin NM , et al. A Dietary fiber‐deprived gut microbiota degrades the colonic mucus barrier and enhances pathogen susceptibility. Cell. 2016;167(5):1339‐1353 e21. 27863247 10.1016/j.cell.2016.10.043 PMC5131798

[14] Frazier K , Chang EB . Intersection of the gut microbiome and circadian rhythms in metabolism. Trends Endocrinol Metab. 2020;31(1):25‐36. 31677970 10.1016/j.tem.2019.08.013 PMC7308175

[15] Guan B , Tong J , Hao H , et al. Bile acid coordinates microbiota homeostasis and systemic immunometabolism in cardiometabolic diseases. Acta Pharm Sin B. 2022;12(5):2129‐2149. 35646540 10.1016/j.apsb.2021.12.011 PMC9136572

[16] Vaughn BP , Rank KM , Khoruts A . Fecal Microbiota Transplantation: current Status in treatment of GI and liver disease. Clin Gastroenterol Hepatol. 2019;17(2):353‐361. 30055267 10.1016/j.cgh.2018.07.026

[17] Eggerth AH , Gagnon BH . The bacteroides of human feces. J Bacteriol. 1933;25(4):389‐413. 16559622 10.1128/jb.25.4.389-413.1933 PMC533498

[18] Sakamoto M , Benno Y . Reclassification of Bacteroides distasonis, Bacteroides goldsteinii and Bacteroides merdae as Parabacteroides distasonis gen. nov., comb. nov., Parabacteroides goldsteinii comb. nov. and Parabacteroides merdae comb. nov. Int J Syst Evol Microbiol. 2006;56(Pt 7):1599‐1605. 16825636 10.1099/ijs.0.64192-0

[19] Wang K , Liao M , Zhou N , et al. Parabacteroides distasonis alleviates obesity and metabolic dysfunctions via production of succinate and secondary bile acids. Cell Rep. 2019;26(1):222‐235.e5. 30605678 10.1016/j.celrep.2018.12.028

[20] Kverka M , Zakostelska Z , Klimesova K , et al. Oral administration of Parabacteroides distasonis antigens attenuates experimental murine colitis through modulation of immunity and microbiota composition. Clin Exp Immunol. 2011;163(2):250‐259. 21087444 10.1111/j.1365-2249.2010.04286.x PMC3043316

[21] Vallianou NG , Kounatidis D , Tsilingiris D , et al. The Role of next‐generation probiotics in obesity and obesity‐associated disorders: current knowledge and future perspectives. Int J Mol Sci. 2023;24(7):6755. 37047729 10.3390/ijms24076755 PMC10095285

[22] Cuffaro B , Assohoun ALW , Boutillier D , et al. In Vitro Characterization of gut microbiota‐derived commensal strains: selection of Parabacteroides distasonis strains alleviating TNBS‐induced colitis in mice. Cells. 2020;9(9):2104. 32947881 10.3390/cells9092104 PMC7565435

[23] Huang J , Liu D , Wang Y , et al. Ginseng polysaccharides alter the gut microbiota and kynurenine/tryptophan ratio, potentiating the antitumour effect of antiprogrammed cell death 1/programmed cell death ligand 1 (anti‐PD‐1/PD‐L1) immunotherapy. Gut. 2022;71(4):734‐745. 34006584 10.1136/gutjnl-2020-321031 PMC8921579

[24] O'Hara AM , Shanahan F . Gut microbiota: mining for therapeutic potential. Clin Gastroenterol Hepatol. 2007;5(3):274‐284. 17368226 10.1016/j.cgh.2006.12.009

[25] Wollenweber HW , Rietschel ET , Hofstad T , Weintraub A , Lindberg AA . Nature, type of linkage, quantity, and absolute configuration of (3‐hydroxy) fatty acids in lipopolysaccharides from Bacteroides fragilis NCTC 9343 and related strains. J Bacteriol. 1980;144(3):898‐903. 7440508 10.1128/jb.144.3.898-903.1980 PMC294751

[26] Bank NC , Singh V , Rodriguez‐Palacios A . Classification of Parabacteroides distasonis and other Bacteroidetes using O‐ antigen virulence gene: RfbA‐Typing and hypothesis for pathogenic vs. probiotic strain differentiation. Gut Microbes. 2022;14(1):1997293. 35090379 10.1080/19490976.2021.1997293 PMC8803095

[27] Wexler HM , Getty C , Fisher G . The isolation and characterisation of a major outer‐membrane protein from Bacteroides distasonis. J Med Microbiol. 1992;37(3):165‐175. 1325560 10.1099/00222615-37-3-165

[28] Wexler HM . Outer‐membrane pore‐forming proteins in gram‐negative anaerobic bacteria. Clin Infect Dis. 2002;35(Suppl 1):S65‐S71. 12173111 10.1086/341923

[29] Fletcher CM , Coyne MJ , Bentley DL , Villa OF , Comstock LE . Phase‐variable expression of a family of glycoproteins imparts a dynamic surface to a symbiont in its human intestinal ecosystem. Proc Natl Acad Sci USA. 2007;104(7):2413‐8. 10.1073/pnas.0608797104 PMC1892957 17284602

[30] Avall‐Jääskeläinen S , Palva A . Lactobacillus surface layers and their applications. FEMS Microbiol Rev. 2005;29(3):511‐529. 15935509 10.1016/j.femsre.2005.04.003

[31] Ryan A , Lynch M , Smith SM , et al. A role for TLR4 in Clostridium difficile infection and the recognition of surface layer proteins. PLoS Pathog. 2011;7(6):e1002076. 21738466 10.1371/journal.ppat.1002076 PMC3128122

[32] Ðapa T , Leuzzi R , Ng YK , et al. Multiple factors modulate biofilm formation by the anaerobic pathogen Clostridium difficile. J Bacteriol. 2013;195(3):545‐555. 23175653 10.1128/JB.01980-12 PMC3554014

[33] Chamarande J , Cunat L , Caillet C , et al. Surface properties of parabacteroides distasonis and impacts of stress‐induced molecules on its Surface adhesion and biofilm formation capacities. Microorganisms. 2021;27(9):1602. 10.3390/microorganisms9081602 PMC8400631 34442682

[34] Kasper DL . The polysaccharide capsule of Bacteroides fragilis subspecies fragilis: immunochemical and morphologic definition. J Infect Dis. 1976;133(1):79‐87. 1451 10.1093/infdis/133.1.79

[35] Onderdonk AB , Moon NE , Kasper DL , Bartlett JG . Adherence of Bacteroides fragilis in vivo. Infect Immun. 1978;19(3):1083‐7. 10.1128/iai.19.3.1083-1087.1978 PMC422299 640723

[36] An H , Qian C , Huang Y , et al. Functional vulnerability of liver macrophages to capsules defines virulence of blood‐borne bacteria. J Exp Med. 2022;219(4):e20212032. 35258552 10.1084/jem.20212032 PMC8908791

[37] Babb JL , Cummins CS . Encapsulation of Bacteroides species. Infect Immun. 1978;19(3):1088‐1091. 640724 10.1128/iai.19.3.1088-1091.1978 PMC422300

[38] Bjornson AB , Bjornson HS , Ashraf M , Lang TJ . Quantitative variability in requirements for opsonization of strains within the Bacteroides fragilis group. J Infect Dis. 1983;148(4):667‐675. 6631060 10.1093/infdis/148.4.667

[39] Nakano V , Piazza RM , Cianciarullo AM , et al. Adherence and invasion of Bacteroidales isolated from the human intestinal tract. Clin Microbiol Infect. 2008;14(10):955‐963. 18828854 10.1111/j.1469-0691.2008.02069.x

[40] Xu J , Mahowald MA , Ley RE , et al. Evolution of symbiotic bacteria in the distal human intestine. PLoS Biol. 2007;5(7):e156. 17579514 10.1371/journal.pbio.0050156 PMC1892571

[41] Rudek W , Haque R . Purification of a mucopolysacharidase from Bacteroides distasonis. J Gen Microbiol. 1980;119(1):211‐5. 10.1099/00221287-119-1-211 7411119

[42] Huang K , Wang MM , Kulinich A , et al. Biochemical characterisation of the neuraminidase pool of the human gut symbiont Akkermansia muciniphila. Carbohydr Res. 2015;415:60‐65. 26340137 10.1016/j.carres.2015.08.001

[43] Fraser AG , Brown R . Neuraminidase production by Bacteroidaceae. J Med Microbiol. 1981;14(1):63‐76. 7463468 10.1099/00222615-14-1-63

[44] Gamage H , Chong RWW , Bucio‐Noble D , et al. Changes in dietary fiber intake in mice reveal associations between colonic mucin O‐glycosylation and specific gut bacteria. Article. Gut Microbes. 2020;12(1):1802209. 32991816 10.1080/19490976.2020.1802209 PMC7781582

[45] Tailford LE , Crost EH , Kavanaugh D , Juge N . Mucin glycan foraging in the human gut microbiome. Front Genet. 2015;6:81. 25852737 10.3389/fgene.2015.00081 PMC4365749

[46] Besanceney‐Webler C , Jiang H , Wang W , Baughn AD , Wu P . Metabolic labeling of fucosylated glycoproteins in Bacteroidales species. Bioorg Med Chem Lett. 2011;21(17):4989‐4992. 21676614 10.1016/j.bmcl.2011.05.038 PMC3156311

[47] Coyne MJ , Reinap B , Lee MM , Comstock LE . Human symbionts use a host‐like pathway for surface fucosylation. Science. 2005;307(5716):1778‐1781. 15774760 10.1126/science.1106469

[48] Nihira T , Suzuki E , Kitaoka M , Nishimoto M , Ohtsubo K , Nakai H . Discovery of beta‐1,4‐D‐mannosyl‐N‐acetyl‐D‐glucosamine phosphorylase involved in the metabolism of N‐glycans. J Biol Chem. 2013;288(38):27366‐27374. 23943617 10.1074/jbc.M113.469080 PMC3779731

[49] Salyers AA , Palmer JK , Wilkins TD . Laminarinase (beta‐glucanase) activity in Bacteroides from the human colon. Appl Environ Microbiol. 1977;33(5):1118‐1124. 879772 10.1128/aem.33.5.1118-1124.1977 PMC170836

[50] Shimizu H , Nakajima M , Miyanaga A , et al. Characterization and structural analysis of a novel exo‐Type enzyme acting on beta‐1,2‐Glucooligosaccharides from Parabacteroides distasonis. Biochemistry. 2018;57(26):3849‐3860. 29763309 10.1021/acs.biochem.8b00385

[51] Tannock GW . Characteristics of Bacteroides isolates from the cecum of conventional mice. Appl Environ Microbiol. 1977;33(4):745‐750. 869524 10.1128/aem.33.4.745-750.1977 PMC170761

[52] Averina OV , Kovtun AS , Polyakova SI , Savilova AM , Rebrikov DV , Danilenko VN . The bacterial neurometabolic signature of the gut microbiota of young children with autism spectrum disorders. J Med Microbiol. 2020;69(4):558‐571. 32213246 10.1099/jmm.0.001178

[53] Avelar KE , Vieira JM , Antunes LC , et al. Antimicrobial resistance of strains of the Bacteroides fragilis group isolated from the intestinal tract of children and adults in Brazil. Int J Antimicrob Agents. 2001;18(2):129‐134. 11516935 10.1016/s0924-8579(01)00354-5

[54] Snydman DR , Jacobus NV , McDermott LA , et al. National survey on the susceptibility of Bacteroides Fragilis Group: report and analysis of trends for 1997–2000. Clin Infect Dis. 2002;35(Suppl 1):S126‐S134. 12173121 10.1086/341934

[55] Vázquez‐López R , Solano‐Gálvez S , Álvarez‐Hernández DA , et al. The Beta‐Lactam resistome expressed by aerobic and anaerobic Bacteria isolated from human feces of healthy donors. Pharmaceuticals (Basel). 2021;14(6):533. 34204872 10.3390/ph14060533 PMC8228550

[56] Boente RF , Ferreira LQ , Falcao LS , et al. Detection of resistance genes and susceptibility patterns in Bacteroides and Parabacteroides strains. Anaerobe. 2010;16(3):190‐194. 20159050 10.1016/j.anaerobe.2010.02.003

[57] Avelar KE , Otsuki K , Vicente AC , et al. Presence of the cfxA gene in Bacteroides distasonis. Res Microbiol. 2003;154(5):369‐374. 12837513 10.1016/S0923-2508(03)00093-7

[58] Malouin F , Lamothe F . The role of beta‐lactamase and the permeability barrier on the activity of cephalosporins against members of the Bacteroides fragilis group. Can J Microbiol. 1987;33(3):262‐266. 3105858 10.1139/m87-044

[59] Wexler HM , Halebian S . Alterations to the penicillin‐binding proteins in the Bacteroides fragilis group: a mechanism for non‐beta‐lactamase mediated cefoxitin resistance. J Antimicrob Chemother. 1990;26(1):7‐20. 10.1093/jac/26.1.7 2211448

[60] Kierzkowska M , Majewska A , Mlynarczyk G . Trends and Impact in antimicrobial resistance among Bacteroides and Parabacteroides species in 2007–2012 compared to 2013–2017. Microb Drug Resist. 2020;26(12):1452‐1457. 32407191 10.1089/mdr.2019.0462

[61] Kierzkowska M , Majewska A , Mlynarczyk G . Transfer of multiple antibiotic resistance between subspecies of Bacteroides fragilis. J Infect Dis. 1979;139(1):97‐101. 108340 10.1093/infdis/139.1.97

[62] Kierzkowska M , Majewska A , Szymanek‐Majchrzak K , Sawicka‐Grzelak A , Mlynarczyk A , Mlynarczyk G . In vitro effect of clindamycin against Bacteroides and Parabacteroides isolates in Poland. J Glob Antimicrob Resist. 2018;13:49‐52. 29129778 10.1016/j.jgar.2017.11.001

[63] Leng Z , Riley DE , Berger RE , Krieger JN , Roberts MC . Distribution and mobility of the tetracycline resistance determinant tetQ. J Antimicrob Chemother. 1997;40(4):551‐559. 9372425 10.1093/jac/40.4.551

[64] Costerton JW , Lewandowski Z , Caldwell DE , Korber DR , Lappin‐Scott HM . Microbial biofilms. Annu Rev Microbiol. 1995;49:711‐745. 8561477 10.1146/annurev.mi.49.100195.003431

[65] Alauzet C , Cunat L , Wack M , et al. Impact of a Model used to simulate chronic socio‐environmental stressors encountered during spaceflight on murine intestinal microbiota. Int J Mol Sci. 2020;21(21):7863. 33114008 10.3390/ijms21217863 PMC7672645

[66] Zhao Q , Dai M‐Y , Huang R‐Y , et al. Parabacteroides distasonis ameliorates hepatic fibrosis potentially via modulating intestinal bile acid metabolism and hepatocyte pyroptosis in male mice. Nat Commun. 2023;14(1):1829. 37005411 10.1038/s41467-023-37459-z PMC10067939

[67] Su X , Gao Y , Yang R . Gut microbiota‐derived tryptophan metabolites maintain gut and systemic homeostasis. Cells. 2022;11(15):2296. 35892593 10.3390/cells11152296 PMC9330295

[68] Davila A‐M , Blachier F , Gotteland M , et al. Intestinal luminal nitrogen metabolism: role of the gut microbiota and consequences for the host. Pharmacol Res. 2013;68(1):95‐107. 23183532 10.1016/j.phrs.2012.11.005

[69] Han S , Van Treuren W , Fischer CR , et al. A metabolomics pipeline for the mechanistic interrogation of the gut microbiome. Nature. 2021;595(7867):415‐420. 34262212 10.1038/s41586-021-03707-9 PMC8939302

[70] Jilly BJ , Schreckenberger PC , LeBeau LJ . Rapid glutamic acid decarboxylase test for identification of Bacteroides and Clostridium spp. J Clin Microbiol. 1984;19(5):592‐593. 6376535 10.1128/jcm.19.5.592-593.1984 PMC271137

[71] Liang Z , Di N , Li L , Yang D . Gut microbiota alterations reveal potential gut‐brain axis changes in polycystic ovary syndrome. J Endocrinol Invest. 2021;44(8):1727‐1737. 33387350 10.1007/s40618-020-01481-5

[72] Wu LT , Tang ZR , Chen HY , et al. Mutual interaction between gut microbiota and protein/amino acid metabolism for host mucosal immunity and health. Anim Nutr. 2021;7(1):11‐16. 33997326 10.1016/j.aninu.2020.11.003 PMC8110859

[73] Ahmed S , Busetti A , Fotiadou P , et al. In vitro Characterization of gut microbiota‐derived bacterial strains with neuroprotective properties. Front Cell Neurosci. 2019;13:402. 31619962 10.3389/fncel.2019.00402 PMC6763572

[74] Cuffaro B , Assohoun ALW , Boutillier D , et al. Identification of new potential biotherapeutics from human gut microbiota‐derived Bacteria. Microorganisms. 2021;9(3):565. 33803291 10.3390/microorganisms9030565 PMC7998412

[75] Russell WR , Duncan SH , Scobbie L , et al. Major phenylpropanoid‐derived metabolites in the human gut can arise from microbial fermentation of protein. Mol Nutr Food Res. 2013;57(3):523‐535. 23349065 10.1002/mnfr.201200594

[76] Saito Y , Sato T , Nomoto K , Tsuji H . Identification of phenol‐ and p‐cresol‐producing intestinal bacteria by using media supplemented with tyrosine and its metabolites. FEMS Microbiol Ecol. 2018;94(9):fiy125. 29982420 10.1093/femsec/fiy125 PMC6424909

[77] Wikoff WR , Anfora AT , Liu J , et al. Metabolomics analysis reveals large effects of gut microflora on mammalian blood metabolites. Proc Natl Acad Sci USA. 2009;106(10):3698‐3703. 19234110 10.1073/pnas.0812874106 PMC2656143

[78] Agus A , Planchais J , Sokol H . Gut microbiota regulation of tryptophan metabolism in health and disease. Cell Host Microbe. 2018;23(6):716‐724. 29902437 10.1016/j.chom.2018.05.003

[79] Cheng L , Wu H , Cai X , et al. A Gpr35‐tuned gut microbe brain metabolic axis regulates depressive‐like behavior. Cell Host Microbe. 2024;32(2):227‐243.e6. 38198925 10.1016/j.chom.2023.12.009

[80] Liu D , Zhang S , Li S , et al. Indoleacrylic acid produced by Parabacteroides distasonis alleviates type 2 diabetes via activation of AhR to repair intestinal barrier. BMC Biol. 2023;21(1):90. 37072819 10.1186/s12915-023-01578-2 PMC10114473

[81] Andreoli AJ , Ikeda M , Nishizuka Y , Hayaishi O . Quinolinic acid: a precursor to nicotinamide adenine dinucleotide in Escherichia coli. Biochem Biophys Res Commun. 1963;12:92‐97. 14013029 10.1016/0006-291x(63)90241-9

[82] Sun Y , Nie Q , Zhang S , et al. Parabacteroides distasonis ameliorates insulin resistance via activation of intestinal GPR109a. Nat Commun. 2023;14(1):7740. 38007572 10.1038/s41467-023-43622-3 PMC10676405

[83] Montgomery TL , Eckstrom K , Lile KH , et al. Lactobacillus reuteri tryptophan metabolism promotes host susceptibility to CNS autoimmunity. Microbiome. 2022;10(1):198. 36419205 10.1186/s40168-022-01408-7 PMC9685921

[84] Barroso A , Mahler JV , Fonseca‐Castro PH , Quintana FJ . The aryl hydrocarbon receptor and the gut‐brain axis. Cell Mol Immunol. 2021;18(2):259‐268. 33408340 10.1038/s41423-020-00585-5 PMC8027889

[85] Meng D , Sommella E , Salviati E , et al. Indole‐3‐lactic acid, a metabolite of tryptophan, secreted by Bifidobacterium longum subspecies infantis is anti‐inflammatory in the immature intestine. Pediatr Res. 2020;88(2):209‐217. 31945773 10.1038/s41390-019-0740-x PMC7363505

[86] Stockinger B , Shah K , Wincent E . AHR in the intestinal microenvironment: safeguarding barrier function. Nat Rev Gastroenterol Hepatol. 2021;18(8):559‐570. 33742166 10.1038/s41575-021-00430-8 PMC7611426

[87] Pugin B , Barcik W , Westermann P , et al. A wide diversity of bacteria from the human gut produces and degrades biogenic amines. Microb Ecol Health Dis. 2017;28(1):1353881. 28959180 10.1080/16512235.2017.1353881 PMC5614385

[88] Hamana K , Matsuzaki S . Polyamines as a Chemotaxonomic marker in Bacterial systematics. Crit Rev Microbiol. 1992;18(4):261‐283. 1524675 10.3109/10408419209113518

[89] Hamana K , Itoh T , Benno Y , Hayashi H . Polyamine distribution profiles of new members of the phylum Bacteroidetes. J Gen Appl Microbiol. 2008;54(4):229‐236. 18802322 10.2323/jgam.54.229

[90] Burrell M , Hanfrey CC , Murray EJ , Stanley‐Wall NR , Michael AJ . Evolution and multiplicity of arginine decarboxylases in polyamine biosynthesis and essential role in Bacillus subtilis biofilm rormation. J Biol Chem. 2010;285(50):39224‐39238. 20876533 10.1074/jbc.M110.163154 PMC2998088

[91] Nakamura A , Ooga T , Matsumoto M . Intestinal luminal putrescine is produced by collective biosynthetic pathways of the commensal microbiome. Gut Microbes. 2019;10(2):159‐171. 30183487 10.1080/19490976.2018.1494466 PMC6546329

[92] Michael AJ . Polyamine function in archaea and bacteria. J Biol Chem. 2018;293(48):18693‐18701. 30254075 10.1074/jbc.TM118.005670 PMC6290158

[93] Sugiyama Y , Nara M , Sakanaka M , et al. Comprehensive analysis of polyamine transport and biosynthesis in the dominant human gut bacteria: Potential presence of novel polyamine metabolism and transport genes. Int J Biochem Cell Biol. 2017;93:52‐61. 29102547 10.1016/j.biocel.2017.10.015

[94] Matsumoto M , Benno Y . The relationship between microbiota and polyamine concentration in the human intestine: a pilot study. Microbiol Immunol. 2007;51(1):25‐35. 17237596 10.1111/j.1348-0421.2007.tb03887.x

[95] Zhao Q , Huang JF , Cheng Y , et al. Polyamine metabolism links gut microbiota and testicular dysfunction. Microbiome. 2021;9(1):224. 34758869 10.1186/s40168-021-01157-z PMC8582214

[96] Lee K‐A , Kim S‐H , Kim E‐K , et al. Bacterial‐derived uracil as a modulator of mucosal immunity and gut‐microbe homeostasis in Drosophila. Cell. 2013;153(4):797‐811. 23663779 10.1016/j.cell.2013.04.009

[97] Lee JS , Wang RX , Goldberg MS , Clifford GP , Kao DJ , Colgan SP . Microbiota‐sourced purines support wound healing and mucous barrier function. iScience. 2020;23(6):101226. 32554188 10.1016/j.isci.2020.101226 PMC7303675

[98] Lee JS , Wang RX , Alexeev EE , et al. Hypoxanthine is a checkpoint stress metabolite in colonic epithelial energy modulation and barrier function. J Biol Chem. 2018;293(16):6039‐6051. 29487135 10.1074/jbc.RA117.000269 PMC5912467

[99] Kuang J , Wang J , Li Y , et al. Hyodeoxycholic acid alleviates non‐alcoholic fatty liver disease through modulating the gut‐liver axis. Cell Metab. 2023;35(10):1752‐1766.e8. 37591244 10.1016/j.cmet.2023.07.011

[100] de Aguiar Vallim TQ , Tarling EJ , Edwards PA . Pleiotropic roles of bile acids in metabolism. Cell Metab. 2013;17(5):657‐669. 23602448 10.1016/j.cmet.2013.03.013 PMC3654004

[101] Song X , Sun X , Oh SF , et al. Microbial bile acid metabolites modulate gut RORγ regulatory T cell homeostasis. Nature. 2020;577(7790):410‐415. 31875848 10.1038/s41586-019-1865-0 PMC7274525

[102] Ridlon JM , Kang DJ , Hylemon PB . Bile salt biotransformations by human intestinal bacteria. J Lipid Res. 2006;47(2):241‐259. 16299351 10.1194/jlr.R500013-JLR200

[103] Choudhuri S , Klaassen CD . Molecular regulation of bile acid homeostasis. Drug Metab Dispos. 2022;50(4):425‐455. 34686523 10.1124/dmd.121.000643

[104] Ridlon JM , Devendran S , Alves JM , et al. The ‘in vivo lifestyle’ of bile acid 7α‐dehydroxylating bacteria: comparative genomics, metatranscriptomic, and bile acid metabolomics analysis of a defined microbial community in gnotobiotic mice. Gut Microbes. 2020;11(3):381‐404. 31177942 10.1080/19490976.2019.1618173 PMC7524365

[105] Narushima S , Itoha K , Miyamoto Y , et al. Deoxycholic acid formation in gnotobiotic mice associated with human intestinal bacteria. Lipids. 2006;41(9):835‐843. 17152920 10.1007/s11745-006-5038-1

[106] Narushima S , Itoh K , Takamine F , Uchida K . Absence of cecal secondary bile acids in gnotobiotic mice associated with two human intestinal bacteria with the ability to dehydroxylate bile acids in vitro. Microbiol Immunol. 1999;43(9):893‐7. 10.1111/j.1348-0421.1999.tb01224.x 10553682

[107] Takamine F , Imamura T . 7 beta‐dehydroxylation of 3,7‐dihydroxy bile acids by a Eubacterium species strain C‐25 and stimulation of 7 beta‐dehydroxylation by Bacteroides distasonis strain K‐5. Microbiol Immunol. 1985;29(12):1247‐1252. 3831723 10.1111/j.1348-0421.1985.tb00915.x

[108] Winston JA , Theriot CM . Diversification of host bile acids by members of the gut microbiota. Gut Microbes. 2020;11(2):158‐171. 31595814 10.1080/19490976.2019.1674124 PMC7053883

[109] Li M , Wang S , Li Y , et al. Gut microbiota‐bile acid crosstalk contributes to the rebound weight gain after calorie restriction in mice. Nat Commun. 2022;13(1):2060. 35440584 10.1038/s41467-022-29589-7 PMC9018700

[110] Hang S , Paik D , Yao L , et al. Bile acid metabolites control T(H)17 and T(reg) cell differentiation. Nature. 2019;576(7785):143‐148. 31776512 10.1038/s41586-019-1785-z PMC6949019

[111] Paik D , Yao L , Zhang Y , et al. Human gut bacteria produce ΤΗ17‐modulating bile acid metabolites. Nature. 2022;603(7903):907‐912. 35296854 10.1038/s41586-022-04480-z PMC9132548

[112] Sato Y , Atarashi K , Plichta DR , et al. Novel bile acid biosynthetic pathways are enriched in the microbiome of centenarians. Nature. 2021;599(7885):458‐464. 34325466 10.1038/s41586-021-03832-5

[113] Sun H , Guo Y , Wang H , et al. Gut commensal Parabacteroides distasonis alleviates inflammatory arthritis. Gut. 2023;72(9):1664‐1677. 36604114 10.1136/gutjnl-2022-327756

[114] Liu C , Du M‐X , Xie L‐S , et al. Gut commensal Christensenella minuta modulates host metabolism via acylated secondary bile acids. Nat Microbiol. 2024;9(2):434‐450. 38233647 10.1038/s41564-023-01570-0

[115] Yan P , Sun Y , Luo J , Liu X , Wu J , Miao Y . Integrating the serum proteomic and fecal metaproteomic to analyze the impacts of overweight/obesity on IBD: a pilot investigation. Clin Proteomics. 2023;20(1):6. 36759757 10.1186/s12014-023-09396-y PMC9909917

[116] Gaifem J , Mendes‐Frias A , Wolter M , et al. Akkermansia muciniphila and Parabacteroides distasonis synergistically protect from colitis by promoting ILC3 in the gut. mBio. 2024;15(4):e0007824. 38470269 10.1128/mbio.00078-24 PMC11210198

[117] Gomez‐Nguyen A , Basson AR , Dark‐Fleury L , et al. Parabacteroides distasonis induces depressive‐like behavior in a mouse model of Crohn's disease. Brain Behav Immun. 2021;98:245‐250. 34403735 10.1016/j.bbi.2021.08.218 PMC9217177

[118] Ma M , Fu T , Wang Y , et al. Polysaccharide from edible alga Enteromorpha clathrata Improves ulcerative colitis in association with increased abundance of Parabacteroides spp. in the gut microbiota of dextran sulfate Sodium‐Fed Mice. Mar Drugs. 2022;20(12):764. 36547911 10.3390/md20120764 PMC9786108

[119] Dziarski R , Park SY , Kashyap DR , Dowd SE , Gupta D . Pglyrp‐regulated gut microflora prevotella falsenii, Parabacteroides distasonis and Bacteroides eggerthii enhance and alistipes finegoldii attenuates colitis in mice. PLoS One. 2016;11(1):e0146162. 26727498 10.1371/journal.pone.0146162 PMC4699708

[120] Lopetuso LR , Petito V , Graziani C , et al. Gut Microbiota in health, diverticular disease, irritable bowel syndrome, and inflammatory bowel diseases: time for microbial marker of gastrointestinal disorders. Dig Dis. 2018;36(1):56‐65. 28683448 10.1159/000477205

[121] Nagayama M , Yano T , Atarashi K , et al. TH1 cell‐inducing Escherichia coli strain identified from the small intestinal mucosa of patients with Crohn's disease. Gut Microbes. 2020;12(1):1788898. 10.1080/19490976.2020.1788898 PMC7524366 32691669

[122] Yang F , Kumar A , Davenport KW , et al. Complete genome sequence of a Parabacteroides distasonis strain (CavFT hAR46) isolated from a gut wall‐cavitating microlesion in a patient with severe crohn's disease. Microbiol Resour Announc. 2019; 8(36):e00585‐519. 10.1128/MRA.00585-19 PMC6728636 31488526

[123] Wang N , Fang JY . Fusobacterium nucleatum, a key pathogenic factor and microbial biomarker for colorectal cancer. Trends Microbiol. 2023;31(2):159‐172. 36058786 10.1016/j.tim.2022.08.010

[124] Yang J , Wei H , Zhou Y , et al. High‐fat diet promotes colorectal tumorigenesis through modulating gut microbiota and metabolites. Gastroenterology. 2022;162(1):135‐149.e2. 34461052 10.1053/j.gastro.2021.08.041

[125] Zhu H , Li M , Bi D , et al. Fusobacterium nucleatum promotes tumor progression in KRAS p.G12D‐mutant colorectal cancer by binding to DHX15. Nat Commun. 2024;15(1):1688. 38402201 10.1038/s41467-024-45572-w PMC10894276

[126] Bai X , Wei H , Liu W , et al. Cigarette smoke promotes colorectal cancer through modulation of gut microbiota and related metabolites. Gut. 2022;71(12):2439‐2450. 35387878 10.1136/gutjnl-2021-325021 PMC9664112

[127] Gu S , Zaidi S , Hassan MI , et al. Mutated CEACAMs Disrupt transforming growth factor beta signaling and alter the intestinal microbiome to promote colorectal carcinogenesis. Gastroenterology. 2020;158(1):238‐252. 31585122 10.1053/j.gastro.2019.09.023 PMC7124154

[128] Parker KD , Maurya AK , Ibrahim H , et al. Dietary rice bran‐modified human gut microbial consortia confers protection against colon carcinogenesis following fecal transfaunation. Biomedicines. 2021;9(2):144. 33546192 10.3390/biomedicines9020144 PMC7913285

[129] Koh GY , Kane AV , Wu X , Crott JW . Parabacteroides distasonis attenuates tumorigenesis, modulates inflammatory markers and promotes intestinal barrier integrity in azoxymethane‐treated A/J mice. Carcinogenesis. 2020;41(7):909‐917. 32115637 10.1093/carcin/bgaa018

[130] Liang R , Li P , Yang N , et al. Parabacteroides distasonis‐derived outer membrane vesicles enhance antitumor immunity against colon tumors by modulating CXCL10 and CD8+ T cells. Drug Des Devel Ther. 2024;18:1833‐1853. 10.2147/DDDT.S457338 PMC11144014 38828018

[131] Yuan N , Li X , Wang M , et al. Gut Microbiota alteration influences colorectal cancer metastasis to the liver by remodeling the liver immune microenvironment. Gut Liver. 2022;16(4):575‐588. 35318288 10.5009/gnl210177 PMC9289841

[132] Creely SJ , McTernan PG , Kusminski CM , et al. Lipopolysaccharide activates an innate immune system response in human adipose tissue in obesity and type 2 diabetes. Am J Physiol Endocrinol Metab. 2007;292(3):E740‐E747. 17090751 10.1152/ajpendo.00302.2006

[133] Xu H , Wang S , Jiang Y , et al. Poria cocos Polysaccharide ameliorated antibiotic‐associated diarrhea in mice via regulating the homeostasis of the gut microbiota and intestinal mucosal barrier. Int J Mol Sci. 2023;24(2):1423. 36674937 10.3390/ijms24021423 PMC9862632

[134] Mocanu V , Park H , Dang J , et al. Timing of tributyrin supplementation differentially modulates gastrointestinal inflammation and gut microbial recolonization following murine ileocecal resection. Nutrients. 2021;13(6):2069. 34204288 10.3390/nu13062069 PMC8233937

[135] Gervason S , Meleine M , Lolignier S , et al. Antihyperalgesic properties of gut microbiota: parabacteroides distasonis as a new probiotic strategy to alleviate chronic abdominal pain. Pain. 2024;165(5):e39‐e54. 37756665 10.1097/j.pain.0000000000003075

[136] Touw K , Ringus DL , Hubert N , et al. Mutual reinforcement of pathophysiological host‐microbe interactions in intestinal stasis models. Physiol Rep. 2017;5(6):e13182. 28320888 10.14814/phy2.13182 PMC5371559

[137] Li M , Liu S , Wang M , et al. Gut microbiota dysbiosis associated with bile acid metabolism in neonatal cholestasis disease. Sci Rep. 2020;10(1):7686. 32377002 10.1038/s41598-020-64728-4 PMC7203226

[138] Chiang JYL , Ferrell JM . Bile acid metabolism in liver pathobiology. Gene Expr. 2018;18(2):71‐87. 29325602 10.3727/105221618X15156018385515 PMC5954621

[139] Wang H , Guo Y , Han W , et al. Tauroursodeoxycholic acid improves nonalcoholic fatty liver disease by regulating gut microbiota and bile acid metabolism. J Agric Food Chem. 2024;72(36):20194‐20210. 39193771 10.1021/acs.jafc.4c04630

[140] Wang L , Zheng W , Sun Y , et al. Fucoidan ameliorates alcohol‐induced liver injury in mice through Parabacteroides distasonis‐mediated regulation of the gut‐liver axis. Int J Biol Macromol. 2024;279(Pt 3):135309. 39236962 10.1016/j.ijbiomac.2024.135309

[141] Yanavich C , Perazzo H , Li F , et al. A pilot study of microbial signatures of liver disease in those with HIV mono‐infection in Rio de Janeiro, Brazil. AIDS. 2022;36(1):49‐58. 34873092 10.1097/QAD.0000000000003084 PMC8667204

[142] Kwan S‐Y , Jiao J , Joon A , et al. Gut microbiome features associated with liver fibrosis in Hispanics, a population at high risk for fatty liver disease. Hepatology. 2022;75(4):955‐967. 34633706 10.1002/hep.32197 PMC8930512

[143] Liang H , Song H , Zhang X , et al. Metformin attenuated sepsis‐related liver injury by modulating gut microbiota. Emerg Microbes Infect. 2022;11(1):815‐828. 35191819 10.1080/22221751.2022.2045876 PMC8928825

[144] Collins SL , Stine JG , Bisanz JE , Okafor CD , Patterson AD . Bile acids and the gut microbiota: metabolic interactions and impacts on disease. Nat Rev Microbiol. 2023;21(4):236‐247. 36253479 10.1038/s41579-022-00805-x PMC12536349

[145] Li X , Wu J , Kang Y , et al. Yeast mannoproteins are expected to be a novel potential functional food for attenuation of obesity and modulation of gut microbiota. Front Nutr. 2022;9:1019344. 36313084 10.3389/fnut.2022.1019344 PMC9614242

[146] He K , Hu Y , Ma H , et al. Rhizoma Coptidis alkaloids alleviate hyperlipidemia in B6 mice by modulating gut microbiota and bile acid pathways. Biochim Biophys Acta. 2016;1862(9):1696‐1709. 27287254 10.1016/j.bbadis.2016.06.006

[147] Moran‐Ramos S , Cerqueda‐García D , López‐Contreras B , et al. A metagenomic study identifies a Prevotella copri enriched microbial profile associated with non‐alcoholic steatohepatitis in subjects with obesity. J Gastroenterol Hepatol. 2023;38(5):791‐799. 36807933 10.1111/jgh.16147

[148] Wei W , Wong CC , Jia Z , et al. Parabacteroides distasonis uses dietary inulin to suppress NASH via its metabolite pentadecanoic acid. Nat Microbiol. 2023;8(8):1534‐1548. 37386075 10.1038/s41564-023-01418-7 PMC10390331

[149] Tarantino G , Sinatti G , Citro V , Santini S, Jr. , Balsano C . Sarcopenia, a condition shared by various diseases: can we alleviate or delay the progression? Intern Emerg Med. 2023;18(7):1887‐1895. 37490203 10.1007/s11739-023-03339-z PMC10543607

[150] Zamboni M , Rubele S , Rossi AP . Sarcopenia and obesity. Curr Opin Clin Nutr Metab Care. 2019;22(1):13‐19. 30461451 10.1097/MCO.0000000000000519

[151] Mai X , Yang S , Chen Q , Chen K . Gut microbial composition is altered in sarcopenia: A systematic review and meta‐analysis of clinical studies. PLoS One. 2024;19(8):e0308360. 39106230 10.1371/journal.pone.0308360 PMC11302912

[152] Lapauw L , Rutten A , Dupont J , et al. Associations between gut microbiota and sarcopenia or its defining parameters in older adults: A systematic review. J Cachexia Sarcopenia Muscle. 2024. 10.1002/jcsm.13569 PMC11634501 39192550

[153] Wang T , Zhou D , Hong Z . Adipose tissue in older individuals: a contributing factor to sarcopenia. Metabolism. 2024;160:155998. 39128607 10.1016/j.metabol.2024.155998

[154] Chawla N , Zengin Z , K Lee , et al. Metagenomic analysis of the gut microbiome in metastatic renal cell carcinoma patients with sarcopenia. IKCS North America. November 4–5, 2022;2022: Abstract 45.

[155] Lackner C , Tiniakos D . Fibrosis and alcohol‐related liver disease. J Hepatol. 2019;70(2):294‐304. 30658730 10.1016/j.jhep.2018.12.003

[156] Zhang H , Li C , Han L , et al. MUP1 mediates urolithin A alleviation of chronic alcohol‐related liver disease via gut‐microbiota‐liver axis. Gut Microbes. 2024;16(1):2367342. 38889450 10.1080/19490976.2024.2367342 PMC11188796

[157] Mortha A , Chudnovskiy A , Hashimoto D , et al. Microbiota‐dependent crosstalk between macrophages and ILC3 promotes intestinal homeostasis. Science. 2014;343(6178):1249288. 24625929 10.1126/science.1249288 PMC4291125

[158] Shi N , Li N , Duan X , Niu H . Interaction between the gut microbiome and mucosal immune system. Mil Med Res. 2017;4:14. 28465831 10.1186/s40779-017-0122-9 PMC5408367

[159] Lehmann FM , von Burg N , Ivanek R , et al. Microbiota‐induced tissue signals regulate ILC3‐mediated antigen presentation. Nat Commun. 2020;11(1):1794. 32286285 10.1038/s41467-020-15612-2 PMC7156681

[160] Paulos CM , Wrzesinski C , Kaiser A , et al. Microbial translocation augments the function of adoptively transferred self/tumor‐specific CD8+ T cells via TLR4 signaling. J Clin Invest. 2007;117(8):2197‐2204. 17657310 10.1172/JCI32205 PMC1924500

[161] Gopalakrishnan V , Spencer CN , Nezi L , et al. Gut microbiome modulates response to anti‐PD‐1 immunotherapy in melanoma patients. Science. 2018;359(6371):97‐103. 29097493 10.1126/science.aan4236 PMC5827966

[162] Sivan A , Corrales L , Hubert N , et al. Commensal Bifidobacterium promotes antitumor immunity and facilitates anti‐PD‐L1 efficacy. Science. 2015;350(6264):1084‐1089. 26541606 10.1126/science.aac4255 PMC4873287

[163] Viaud S , Saccheri F , Mignot G , et al. The intestinal microbiota modulates the anticancer immune effects of cyclophosphamide. Science. 2013;342(6161):971‐976. 24264990 10.1126/science.1240537 PMC4048947

[164] Tanoue T , Morita S , Plichta DR , et al. A defined commensal consortium elicits CD8 T cells and anti‐cancer immunity. Nature. 2019;565(7741):600‐605. 30675064 10.1038/s41586-019-0878-z

[165] Gao J , Shi LZ , Zhao H , et al. Loss of IFN‐γ pathway genes in tumor cells as a mechanism of resistance to anti‐CTLA‐4 therapy. Cell. 2016;167(2):397‐404.e9. 27667683 10.1016/j.cell.2016.08.069 PMC5088716

[166] Cekanaviciute E , Yoo BB , Runia TF , et al. Gut bacteria from multiple sclerosis patients modulate human T cells and exacerbate symptoms in mouse models. Proc Natl Acad Sci USA. 2017;114(40):10713‐10718. 28893978 10.1073/pnas.1711235114 PMC5635915

[167] Wang B , Qiu Y , Xie M , et al. Gut microbiota Parabacteroides distasonis enchances the efficacy of immunotherapy for bladder cancer by activating anti‐tumor immune responses. BMC Microbiol. 2024;24(1):237. 38961326 10.1186/s12866-024-03372-8 PMC11221038

[168] Chamarande J , Cunat L , Pavlov N , Alauzet C , Cailliez‐Grimal C. Parabacteroides distasonis properties linked to the selection of new biotherapeutics. Nutrients. 2022;14(19):4176. 36235828 10.3390/nu14194176 PMC9572384

[169] Hiippala K , Kainulainen V , Suutarinen M , et al. Isolation of anti‐inflammatory and epithelium reinforcing Bacteroides and Parabacteroides Spp. from a healthy fecal donor. Nutrients. 2020;12(4):935. 32230951 10.3390/nu12040935 PMC7230855

[170] Buckner CM , Moir S , Kardava L , et al. CXCR4/IgG‐expressing plasma cells are associated with human gastrointestinal tissue inflammation. J Allergy Clin Immunol. 2014;133(6):1676‐85.e5. 24373354 10.1016/j.jaci.2013.10.050 PMC4040303

[171] Brīvība M , Silamiķele L , Kalniņa I , et al. Metformin targets intestinal immune system signaling pathways in a high‐fat diet‐induced mouse model of obesity and insulin resistance. Front Endocrinol (Lausanne). 2023;14:1232143. 37795356 10.3389/fendo.2023.1232143 PMC10546317

[172] Ouyang W . Distinct roles of IL‐22 in human psoriasis and inflammatory bowel disease. Cytokine Growth Factor Rev. 2010;21(6):435‐441. 21106435 10.1016/j.cytogfr.2010.10.007

[173] Bao Z , Wei R , Zheng X , et al. Landscapes of gut microbiome and bile acid signatures and their interaction in HBV‐associated acute‐on‐chronic liver failure. Front Microbiol. 2023;14:1185993. 37275140 10.3389/fmicb.2023.1185993 PMC10233926

[174] Ma C , Yuan D , Renaud SJ , et al. Chaihu‐shugan‐san alleviates depression‐like behavior in mice exposed to chronic unpredictable stress by altering the gut microbiota and levels of the bile acids hyocholic acid and 7‐ketoDCA. Front Pharmacol. 2022;13:1040591. 36339629 10.3389/fphar.2022.1040591 PMC9627339

[175] Cuffaro B , Boutillier D , Desramaut J , et al. Characterization of two Parabacteroides distasonis candidate strains as new live biotherapeutics against obesity. Cells. 2023;12(9):1260. 37174660 10.3390/cells12091260 PMC10177344

[176] Zhao Q , Yu J , Zhou H , et al. Intestinal dysbiosis exacerbates the pathogenesis of psoriasis‐like phenotype through changes in fatty acid metabolism. Signal Transduct Target Ther. 2023;8(1):40. 36710269 10.1038/s41392-022-01219-0 PMC9884668

[177] Pegg AE . The function of spermine. IUBMB Life. 2014;66(1):8‐18. 24395705 10.1002/iub.1237

[178] Blake KJ , Baral P , Voisin T , et al. Staphylococcus aureus produces pain through pore‐forming toxins and neuronal TRPV1 that is silenced by QX‐314. Nat Commun. 2018;9(1):37. 29295977 10.1038/s41467-017-02448-6 PMC5750211

[179] Ma F , Li Z , Liu H , et al. Dietary‐timing‐induced gut microbiota diurnal oscillations modulate inflammatory rhythms in rheumatoid arthritis. Cell Metab. 2024;S1550‐4131(24)00334‐6. 10.1016/j.cmet.2024.08.007 39260371

[180] Kaur AP , Bhardwaj S , Dhanjal DS , et al. Plant prebiotics and their role in the amelioration of diseases. Biomolecules. 2021;11(3):440. 33809763 10.3390/biom11030440 PMC8002343

[181] Zhu L , Li S , Zheng W , Ni W , Cai M , Liu H . Targeted modulation of gut microbiota by traditional Chinese medicine and natural products for liver disease therapy. Front Immunol. 2023;14:1086078. 36817459 10.3389/fimmu.2023.1086078 PMC9933143

[182] Salvatore S , Battigaglia MS , Murone E , Dozio E , Pensabene L , Agosti M . Dietary fibers in healthy children and in pediatric gastrointestinal disorders: A practical guide. Nutrients. 2023;15(9):2208. 37432354 10.3390/nu15092208 PMC10180776

[183] Lopez‐Santamarina A , Cardelle‐Cobas A , Del Carmen Mondragon A , Sinisterra‐Loaiza L , Miranda JM , Cepeda A . Evaluation of the potential prebiotic effect of Himanthalia elongata, an Atlantic brown seaweed, in an in vitro model of the human distal colon. Food Res Int. 2022;156:111156. 35651022 10.1016/j.foodres.2022.111156

[184] Lv K , Yuan Q , Li H , et al. Chlorella pyrenoidosa polysaccharides as a prebiotic to modulate gut microbiota: physicochemical properties and fermentation characteristics in vitro. Foods. 2022;11(5):725. 35267359 10.3390/foods11050725 PMC8908982

[185] An C , Kuda T , Yazaki T , Takahashi H , Kimura B . FLX pyrosequencing analysis of the effects of the brown‐algal fermentable polysaccharides alginate and laminaran on rat cecal microbiotas. Appl Environ Microbiol. 2013;79(3):860‐866. 23183985 10.1128/AEM.02354-12 PMC3568576

[186] Liu Z , Zhang Y , Ai C , et al. Gut microbiota response to sulfated sea cucumber polysaccharides in a differential manner using an in vitro fermentation model. Food Res Int. 2021;148:110562. 34507721 10.1016/j.foodres.2021.110562

[187] Liu Z , Hu Y , Tao X , et al. Metabolites of sea cucumber sulfated polysaccharides fermented by Parabacteroides distasonis and their effects on cross‐feeding. Food Res Int. 2023;167:112633. 37087229 10.1016/j.foodres.2023.112633

[188] Thirion F , Da Silva K , Plaza Oñate F , et al. Diet Supplementation with NUTRIOSE, a resistant dextrin, increases the abundance of Parabacteroides distasonis in the human gut. Mol Nutr Food Res. 2022;66(11):e2101091. 35312171 10.1002/mnfr.202101091 PMC9287035

[189] J Abell GC , Christophersen CT , McOrist AL , Clarke JM . Dietary resistant and butyrylated starches have different effects on the faecal bacterial flora of azoxymethane‐treated rats. Br J Nutr. 2011;105(10):1480‐1485. 21255474 10.1017/S0007114510005349

[190] Clarke JM , Topping DL , Christophersen CT , et al. Butyrate esterified to starch is released in the human gastrointestinal tract. Am J Clin Nutr. 2011;94(5):1276‐1283. 21940597 10.3945/ajcn.111.017228

[191] Cai W , Xu J , Li G , et al. Ethanol extract of propolis prevents high‐fat diet‐induced insulin resistance and obesity in association with modulation of gut microbiota in mice. Food Res Int. 2020;130:108939. 32156386 10.1016/j.foodres.2019.108939

[192] Liu Z , Chen Q , Zhang C , Ni L . Comparative study of the anti‐obesity and gut microbiota modulation effects of green tea phenolics and their oxidation products in high‐fat‐induced obese mice. Food Chem. 2022;367:130735. 34365247 10.1016/j.foodchem.2021.130735

[193] Polimeno L , Barone M , Mosca A , et al. Soy metabolism by gut microbiota from patients with precancerous intestinal lesions. Microorganisms. 2020;8(4):469. 32218321 10.3390/microorganisms8040469 PMC7232402

[194] Song H , Shen X , Chu Q , Zheng X . Pomegranate fruit pulp polyphenols reduce diet‐induced obesity with modulation of gut microbiota in mice. J Sci Food Agric. 2022;102(5):1968‐1977. 34514612 10.1002/jsfa.11535

[195] Huang Y , Zheng Y , Yang F , et al. Lycium barbarum Glycopeptide prevents the development and progression of acute colitis by regulating the composition and diversity of the gut microbiota in mice. Front Cell Infect Microbiol. 2022;12:921075. 36017369 10.3389/fcimb.2022.921075 PMC9395742

[196] Zhang R , Wu J , Lei Y , et al. Oregano essential oils promote rumen digestive ability by modulating epithelial development and microbiota composition in beef cattle. Front Nutr. 2021;8:722557. 34859026 10.3389/fnut.2021.722557 PMC8631176

[197] Wang T , Zhang C , Li H , et al. The underlying rationality of Chinese medicine herb pair Coptis chinensis and Dolomiaea souliei: From the perspective of metabolomics and intestinal function. J Ethnopharmacol. 2022;289:115065. 35122977 10.1016/j.jep.2022.115065

[198] Xu Y , Wang N , Tan H‐Y , et al. Panax notoginseng saponins modulate the gut microbiota to promote thermogenesis and beige adipocyte reconstruction via leptin‐mediated AMPKα/STAT3 signaling in diet‐induced obesity. Theranostics. 2020;10(24):11302‐11323. 33042284 10.7150/thno.47746 PMC7532683

[199] Jadhav A , Jagtap S , Vyavahare S , Sharbidre A , Kunchiraman B . Reviewing the potential of probiotics, prebiotics and synbiotics: advancements in treatment of ulcerative colitis. Front Cell Infect Microbiol. 2023;13:1268041. 38145046 10.3389/fcimb.2023.1268041 PMC10739422

[200] Alam Z , Shang X , Effat K , et al. The potential role of prebiotics, probiotics, and synbiotics in adjuvant cancer therapy especially colorectal cancer. J Food Biochem. 2022;46(10):e14302. 35816322 10.1111/jfbc.14302

[201] Wu Y , Li Y , Zheng Q , Li L . The Efficacy of Probiotics, Prebiotics, synbiotics, and fecal microbiota transplantation in irritable bowel syndrome: a systematic review and network meta‐analysis. Nutrients. 2024;16(13):2114. 38999862 10.3390/nu16132114 PMC11243554

[202] Aron‐Wisnewsky J , Warmbrunn MV , Nieuwdorp M , Clément K . Nonalcoholic fatty liver disease: modulating gut microbiota to improve severity? Gastroenterology. 2020;158(7):1881‐1898. 32044317 10.1053/j.gastro.2020.01.049

[203] Guardamagna M , Berciano‐Guerrero M‐A , Villaescusa‐González B , et al. Gut microbiota and therapy in metastatic melanoma: focus on MAPK pathway inhibition. Int J Mol Sci. 2022;23(19):11990. 36233289 10.3390/ijms231911990 PMC9569448

[204] Cammarota G , Ianiro G , Tilg H , et al. European consensus conference on faecal microbiota transplantation in clinical practice. Gut. 2017;66(4):569‐580. 28087657 10.1136/gutjnl-2016-313017 PMC5529972

[205] Wang Y , Hunt A , Danziger L , Drwiega EN . A Comparison of currently available and investigational fecal microbiota transplant products for pecurrent Clostridioides difficile infection. Antibiotics (Basel). 2024;13(5):436. 38786164 10.3390/antibiotics13050436 PMC11117328

[206] Costello SP , Hughes PA , Waters O , et al. Effect of fecal microbiota transplantation on 8‐Week remission in patients with ulcerative colitis: a randomized clinical trial. JAMA. 2019;321(2):156‐164. 30644982 10.1001/jama.2018.20046 PMC6439766

[207] Ahn SB , Jun DW , Kang B‐K , Lim JH , Lim S , Chung M‐J. Randomized, double‐blind, placebo‐controlled study of a multispecies probiotic mixture in nonalcoholic fatty liver disease. Sci Rep. 2019;9(1):5688. 30952918 10.1038/s41598-019-42059-3 PMC6450966

[208] El‐Salhy M , Hatlebakk JG , Gilja OH , Bråthen Kristoffersen A , Hausken T . Efficacy of faecal microbiota transplantation for patients with irritable bowel syndrome in a randomised, double‐blind, placebo‐controlled study. Gut. 2020;69(5):859‐867. 31852769 10.1136/gutjnl-2019-319630 PMC7229896

[209] West NP , Christophersen CT , Pyne DB , et al. Butyrylated starch increases colonic butyrate concentration but has limited effects on immunity in healthy physically active individuals. Exerc Immunol Rev. 2013;19:102‐119. 23977723

[210] Le Leu RK , Winter JM , Christophersen CT , et al. Butyrylated starch intake can prevent red meat‐induced O6‐methyl‐2‐deoxyguanosine adducts in human rectal tissue: a randomised clinical trial. Br J Nutr. 2015;114(2):220‐230. 26084032 10.1017/S0007114515001750 PMC4531472

[211] Kiewiet MBG , Elderman ME , El Aidy S , et al. Flexibility of gut microbiota in ageing individuals during dietary fiber long‐chain inulin intake. Mol Nutr Food Res. 2021;65(4):e2000390. 33369019 10.1002/mnfr.202000390 PMC8138623

[212] Hu X , Xia K , Dai M , et al. Intermittent fasting modulates the intestinal microbiota and improves obesity and host energy metabolism. NPJ Biofilms Microbiomes. 2023;9(1):19. 37029135 10.1038/s41522-023-00386-4 PMC10081985

[213] Zhou J , Wu X , Xiang T , et al. Dynamical alterations of brain function and gut microbiome in weight loss. Front Cell Infect Microbiol. 2023;13:1269548. 38173792 10.3389/fcimb.2023.1269548 PMC10761423

[214] García‐Bayona L , Comstock LE . Streamlined Genetic Manipulation of diverse Bacteroides and Parabacteroides isolates from the human gut microbiota. mBio. 2019;10(4):e01762‐19. 31409684 10.1128/mBio.01762-19 PMC6692515

[215] Żółkiewicz J , Marzec A , Ruszczyński M , Feleszko W . Postbiotics‐a step beyond pre‐ and probiotics. Nutrients. 2020;12(8):2189. 32717965 10.3390/nu12082189 PMC7468815

[216] Koh E , Hwang IY , Lee HL , et al. Engineering probiotics to inhibit Clostridioides difficile infection by dynamic regulation of intestinal metabolism. Nat Commun. 2022;13(1):3834. 35787625 10.1038/s41467-022-31334-z PMC9253155

[217] Beukema M , Faas MM , de Vos P . The effects of different dietary fiber pectin structures on the gastrointestinal immune barrier: impact via gut microbiota and direct effects on immune cells. Exp Mol Med. 2020;52(9):1364‐1376. 32908213 10.1038/s12276-020-0449-2 PMC8080816

[218] Gotteland M , Riveros K , Gasaly N , et al. The pros and cons of using algal polysaccharides as prebiotics. Front Nutr. 2020;7:163. 33072794 10.3389/fnut.2020.00163 PMC7536576

